# Chitosan in regenerative medicine: a translational perspective on biomaterial design

**DOI:** 10.3389/fbioe.2026.1924641

**Published:** 2026-09-25

**Authors:** Adrianna Banach-Kopeć, Lisa Gugler, Karol Staszczyk, Szymon Mania, Agnes S. Klar, Robert Tylingo

**Affiliations:** 1 Department of Chemistry, Technology and Biotechnology of Food, Faculty of Chemistry, Gdańsk University of Technology, Gdansk, Poland; 2 Tissue Biology Research Unit, Department of Surgery, University Children’s Hospital Zurich, Zurich, Switzerland; 3 University of Zurich, Zurich, Switzerland

**Keywords:** biomaterial translation, biomaterials, chitosan, regenerative medicine, tissue engineering

## Abstract

Chitosan is an aminopolysaccharide platform with broad relevance to regenerative medicine because its charge density, solubility, degradation, processability, and biological interactions can be adjusted through source selection, deacetylation chemistry, molecular-weight control, derivatization, and formulation. However, this tunability is accompanied by substantial source- and process-dependent variability, which represents a central translational challenge: materials labelled as chitosan can differ markedly in degree of deacetylation, molecular weight, pattern of acetylation, polydispersity, purity, endotoxin and β-glucan burden, residual reagents, sterilization history, and final architecture. This review synthesizes chitosan regenerative biomaterials from a translational design perspective. We examine raw-material provenance, crustacean and fungal sources, extraction and deacetylation routes, physicochemical determinants, medical-grade purity requirements, host-biointerface mechanisms, antimicrobial activity, scaffolds, hydrogels, coatings, bioinks, polymer composites, and functionalization with growth factors, drugs, peptides, and extracellular vesicles. We also address ethical, cell-related, regulatory, and manufacturing issues that arise when chitosan is combined with animal-derived inputs, antibiotics, growth factors, living cells, or extracellular-vesicle preparations. Across applications, the evidence indicates that chitosan should not be treated as a universally biocompatible, universally antimicrobial, or intrinsically regenerative polymer. Rather, it is a tunable, formulation-dependent biomaterial platform whose performance depends on defined chemical attributes, exposure route, biological context, and clinically relevant comparators. Translation will require standardized reporting of degree of deacetylation, molecular weight, acetylation pattern, purity, endotoxin and β-glucan controls, sterilization effects, degradation, release kinetics, post-release bioactivity, and product-specific biological evaluation. Regulatory-aware design, reproducible manufacturing, and mechanism-linked potency assays are essential for moving chitosan-based regenerative systems from promising preclinical constructs toward credible clinical products.

## 1 Introduction

Regenerative medicine integrates cell biology, materials engineering, and targeted therapies to restore tissue and organ function beyond what transplantation can offer. Tissue engineering, which combines cells, biodegradable scaffolds, and growth factors, represents one of its central pillars (Langer and Vacanti, 1993; Dzobo et al., 2018; Gaharwar et al., 2020; Hoang et al., 2025). The driving need is demographic and structural: an aging population and rising chronic disease increase the number of patients with tissue damage, while donor organs remain scarce and long-term care for organ failure burdens health systems (Dzobo et al., 2018; Badawy et al., 2025). Contemporary translation therefore demands more than biological efficacy: reproducibility, manufacturability at scale, and compliance with medical-device and advanced-therapy regulatory frameworks are equally decisive (Hunsberger et al., 2015; Badawy et al., 2025). Accordingly, this review adopts a translational design perspective, treating biomaterials not as inert carriers but as active participants in regeneration whose properties can be tuned to steer repair (Sadtler et al., 2016; Rangel et al., 2024).

### 1.1 Regenerative medicine and the role of biomaterials

Reconstructing living tissue depends on appropriate combinations of cells, biological signals, and biomaterial scaffolds (Langer and Vacanti, 1993; Dzobo et al., 2018). Scaffolds such as porous sponges, membranes, and hydrogels provide a structural framework for cell colonization, deliver bioactive cues, and create a microenvironment conducive to regeneration. An important shift in the field has been the evolution from inert support matrices to “smart” materials that dynamically interact with surrounding tissue by presenting ligands, releasing growth factors at controlled rates, or responding to pH or temperature (Gaharwar et al., 2020; Raheem et al., 2025; Zhang et al., 2025). Such active biomaterials can accelerate angiogenesis, direct stem-cell differentiation, and modulate inflammation, acting simultaneously as extracellular matrix (ECM)-mimicking physical supports and as bioactive modulators of healing (Sadtler et al., 2016; Rangel et al., 2024; Hoang et al., 2025). This dual role increasingly includes controlled bioactive-molecule delivery, immunomodulation, antimicrobial action, and matrix remodeling, including implants with antibacterial or immunomodulatory coatings that prevent infection and steer the host response toward regeneration rather than rejection (Mierzejewska et al., 2026; Abaricia et al., 2021). These principles are summarized schematically in Figure 1.

**FIGURE 1:**
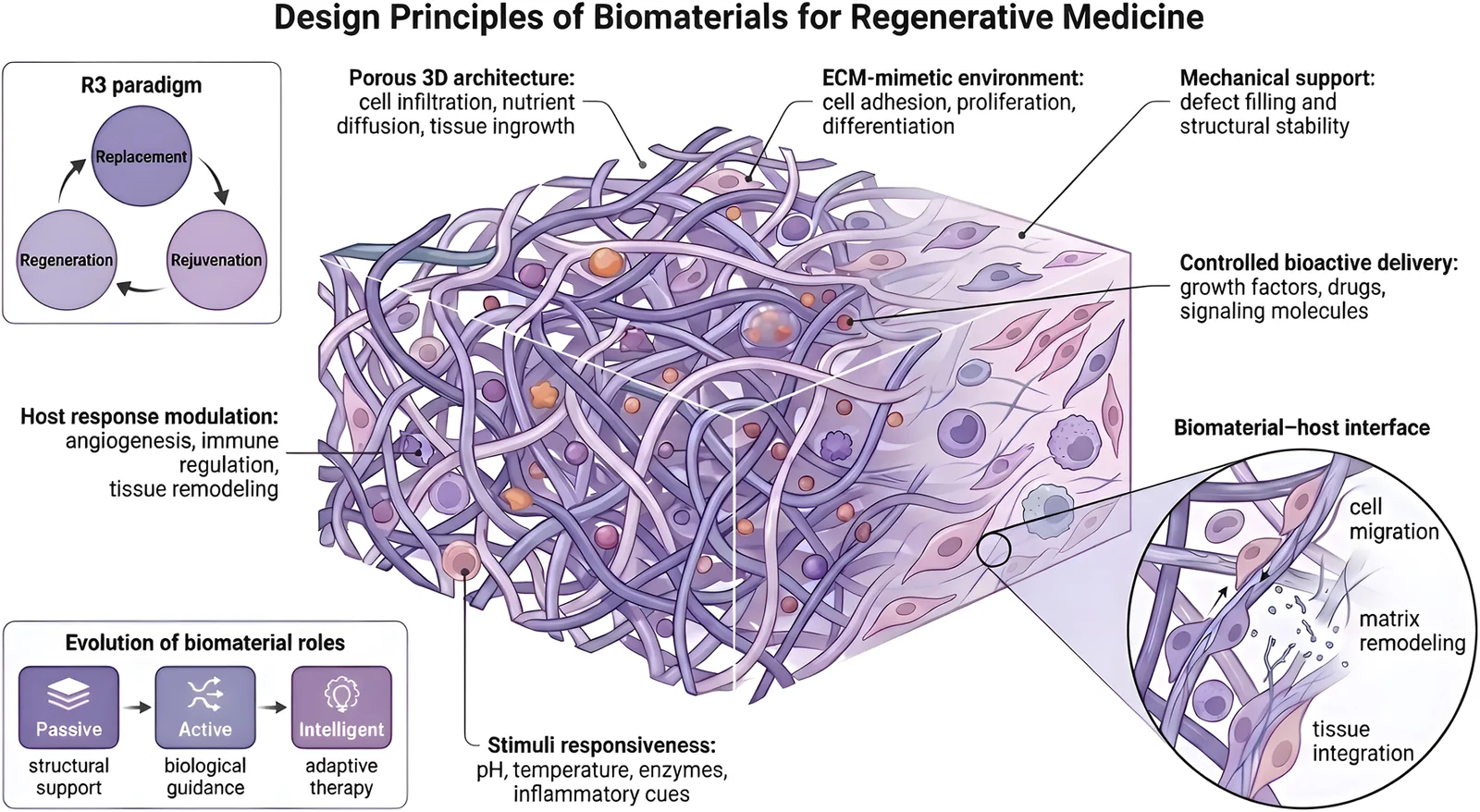
Active biomaterial design for regenerative microenvironments. Modern regenerative biomaterials are designed to act as dynamic regulators of tissue repair rather than passive structural supports. By integrating porous architecture, extracellular-matrix (ECM)-mimetic cues, controlled bioactive delivery, and host-response modulation, they can influence cell infiltration, angiogenesis, inflammation, and matrix remodeling. This figure was prepared with the assistance of FigureLabs.ai.

This narrative critical review synthesizes recent advances in chitosan-based biomaterials from a translational design perspective rather than as a systematic survey. We focus on the relationships between chitosan source, DD, MW, purity, and processing route, on the one hand, and biological functionality and clinical translation potential, on the other, while highlighting key performance-defining features across different formats, including porosity and stiffness in scaffolds, *in situ* gelation in hydrogels, controlled release in nanoscale systems, and printability in bioinks. We also identify the principal remaining gaps: standardized characterization, better reporting of material parameters, control of purity and endotoxin, storage stability, and optimized formulations for regenerative medicine and 3D bioprinting.

### 1.2 Why chitosan?

Among the many biomaterials investigated for regenerative medicine, chitosan has attracted particular attention because of its unique combination of properties (Sukpaita et al., 2021). Chitosan is a natural polysaccharide obtained by deacetylation of chitin, one of the most abundant biopolymers in nature, with commercial chitin being predominantly sourced from crustacean shells (Rodríguez-Vázquez et al., 2015). In defined, soluble, and appropriately protonated formulations, it offers favorable cytocompatibility, biodegradation, and low toxicity (Sultankulov et al., 2019); antibacterial activity against Gram-positive and Gram-negative bacteria, attributed to its protonated amino groups disrupting microbial membranes (Tavaria et al., 2012); and hemostatic activity through erythrocyte aggregation and platelet adhesion/activation, which has supported its use in wound dressings and hemostatic materials (Gheorghiță et al., 2023; Cassano et al., 2024). As detailed in later sections, these properties are formulation- and context-dependent rather than intrinsic to chitosan as a chemical entity. Together with its processability, these context-specific properties make chitosan attractive for wound healing, tissue engineering, and regenerative medicine (Mekhail and Tabrizian, 2014; Kim et al., 2024). Importantly, regulatory recognition for selected uses, including GRAS status in specific contexts, does not constitute general approval for implantable or broader clinical applications, which require separate biological, preclinical, and regulatory evaluation.

Chitosan’s intrinsic chemical modifiability makes it remarkably versatile in biomaterial design (Rodríguez-Vázquez et al., 2015); its multifunctional character and the parameters that determine its biomedical performance are summarized in Figure 2. The glucosamine-based backbone permits adjustment of the degree of deacetylation (DD), introduction of functional groups, crosslinking, and derivatization (Sa et al., 2012), allowing fine-tuning of key physicochemical parameters:

- Surface charge: higher deacetylation yields more protonated amino groups, affecting interactions with cells and proteins (Chevrier et al., 2017);
- Solubility: native chitosan dissolves only in acid, but modifications such as quaternization confer solubility at neutral pH (Kolasa et al., 2022);
- Network-forming ability: chitosan forms networks via chemical crosslinking (e.g., genipin, aldehydes) and ionic gelation with multivalent polyanions such as tripolyphosphate, with properties depending on DD, molecular weight (MW), pH, concentration, and crosslinker (Muzzarelli, 2009; Uddin et al., 2024; Pereira Parchen et al., 2025).

**FIGURE 2:**
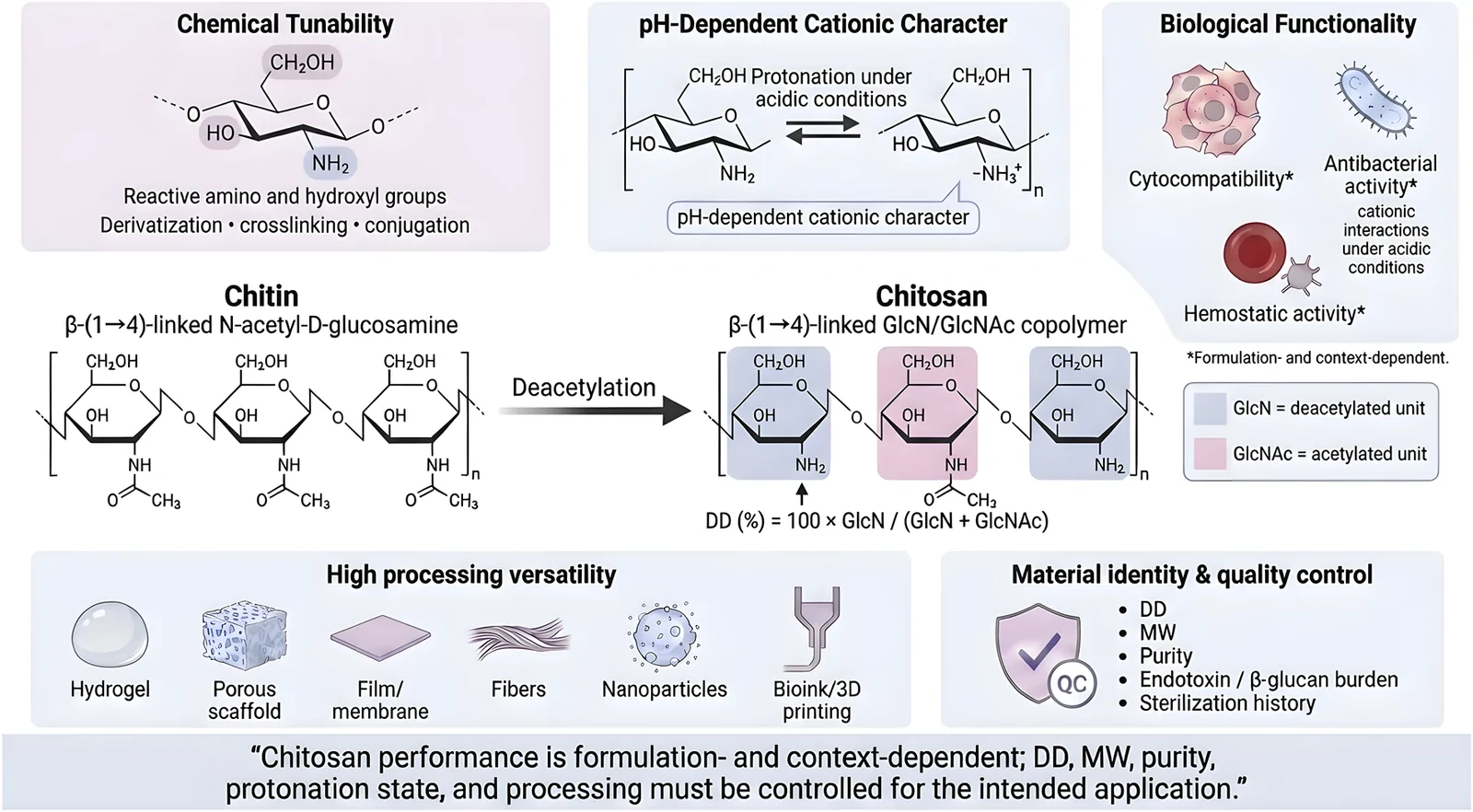
Chitosan as a tunable platform for regenerative biomaterial design. Chitosan combines biodegradation, hemostatic potential, mucoadhesion, formulation-dependent antimicrobial activity, chemical modifiability, and broad processing versatility. Translation into wound dressings, hydrogels, coatings, delivery systems, and bioinks depends on rigorous control of source, DD, MW, purity, formulation, and regulatory requirements. This figure was prepared with the assistance of FigureLabs.ai.

This tunability underlies chitosan’s many biomaterial forms, but safety and compatibility remain product- and context-specific, and effective clinical translation requires early standardization, rigorous quality control of natural variability (MW, DD, purity), safety vetting of any new modification, and integration of polymer chemistry, cell biology, and tissue engineering (Sultankulov et al., 2019; Szulc and Lewandowska, 2023).

### 1.3 The spectrum of chitosan biomaterial forms

Chitosan’s processability allows fabrication into hydrogels, membranes and films, porous sponges, nanofibers, microspheres and nanoparticles, and 3D-printed constructs, each interacting with tissue differently and serving both regenerative medicine and drug delivery (Sukpaita et al., 2021; Rodríguez-Vázquez et al., 2015; Sultankulov et al., 2019; Tavaria et al., 2012). These formats provide the organizing framework for the remainder of this review: hydrogels and injectable systems are examined in the section on hydrogels and injectable materials, implant coatings in the section on coatings and surfaces, and polymer composites and bioinks in the section on functionalization and composites (Huang et al., 2021; Milano et al., 2023).

### 1.4 From promise to practice: Barriers and the need for guidelines

Translating chitosan from *in vitro* promise to clinical use remains difficult for several reasons: raw-material variability in MW and DD undermines reproducibility and scale-up (Acosta-Ferreira et al., 2020; Pratiwi et al., 2023); tightly controlled laboratory parameters are hard to maintain industrially, raising cost (Yadav et al., 2019); and regulatory requirements are stringent, since depending on formulation a chitosan product may be a medical device or an advanced therapy medicinal product (ATMP), each demanding rigorous proof of quality, safety, and efficacy (Hunsberger et al., 2015; Jurczak et al., 2024). For chitosan specifically, raw-material traits drive the entire design chain: DD and MW govern processing and final porosity, mechanics, degradation, and cytocompatibility, so batch-to-batch shifts (e.g., 75% vs. 90% DD) yield divergent, artifact-prone results unless characterization is strictly controlled and consistently reported (Mohamed et al., 2015; Kadyseva et al., 2021; Szymańska and Winnicka, 2015). Harmonized guidelines for raw-material characterization, manufacturing, and standardized functional assays are therefore urgently needed; a minimum reporting set for chitosan biomaterials is proposed in the Conclusions (C.-Eulalio et al., 2020). Crucially, natural origin does not guarantee clinical safety or regulatory acceptance: validation of purity and endotoxin content, assessment of residual immunogenicity, storage stability, and trained research practice are all essential (Alemu et al., 2023; Szymańska and Winnicka, 2015; Aranaz et al., 2021). Variability in raw-material sourcing and supply chains can further exacerbate batch-to-batch differences in chitosan quality. Pharmaceutical- or biomedical-grade material requires additional deproteinization, endotoxin-reduction, and batch-consistency testing beyond industrial-grade chitosan, reinforcing that DD, MW, and purity must be treated as manufacturing-controlled specifications rather than as reported *post hoc* characterization values. Beyond empirical formulation screening, machine-learning-assisted approaches are increasingly applied to biomaterial property prediction and biomanufacturing-process optimization across tissue engineering more broadly (Fu et al., 2025; Gökçekuyu et al., 2024). Chitosan-specific applications of these methods remain rare in the current literature, representing an open methodological gap rather than an established design paradigm for this polymer.

### 1.5 Marketed and clinically investigated chitosan-based products

Chitosan has reached clinical translation, although the maturity of this translation remains strongly application-dependent. The most established examples are concentrated in hemostatic and wound-care devices, whereas fewer chitosan-based platforms have progressed to cartilage repair, peripheral nerve repair, or *in situ* surgical hydrogels (Table 1). Regulatory status and clinical evidence should be considered separately. In the United States, clearance through the 510(k) pathway indicates that the Food and Drug Administration (FDA) has determined a device to be substantially equivalent to a legally marketed predicate device and permits its commercial distribution. Accordingly, products following this pathway are described here as FDA-cleared rather than FDA-approved, and clearance itself should not be interpreted as evidence of clinical superiority (U.S. Food and Drug Administration, 2026a).

**TABLE 1:** Source-verified regulatory-cleared or clinically investigated chitosan-based medical products**.**

Product/platform	Chitosan format/composition	Clinical application	Verified regulatory/clinical status	Representative human evidence/translational information	Sources
HemCon Bandage/ChitoFlex/ChitoGauze	Chitosan-based hemostatic dressings supplied as bandages, flexible strips, or chitosan-containing/coated gauze	External hemorrhage, wound bleeding, and selected vascular-access applications	Multiple FDA 510(k) clearances, including HemCon Bandage K023298, HemCon Bandage/ChitoFlex configurations K080818, and ChitoGauze K090026	Combat-use experience has been reported. Randomized-order crossover study in 50 hemodialysis patients: hemostasis within 4 min in 86% of HemCon-treated sites versus 72% with conventional gauze	(Wedmore et al., 2006; U.S. Food and Drug Administration, 2020; Bachtell et al., 2006)
CELOX family	Chitosan-based hemostatic granules; later configurations incorporate the hemostatic material into gauze	Temporary external control of moderate-to-severe bleeding and penetrating trauma	FDA 510(k) clearances include CELOX Topical Hemostatic Granules K061079, CELOX Rapid Gauze K110386, CELOX Gauze PRO K113560, and CELOX Rapid X-Ray Gauze K230589	RCT, n = 160, penetrating limb injuries: hemostasis within 5 min in 51.3% with Celox-coated gauze versus 32.5% with conventional gauze; time to hemostasis and blood loss were significantly reduced	(U.S. Food and Drug Administration, 2025a; Hatamabadi et al., 2015)
Chito-SAM 100/Chito-SAM Active	Nonwoven fabric derived from chitosan fibers; Chito-SAM Active described in the FDA submission as a 100% chitosan hemostatic dressing	Temporary control of moderate-to-severe external bleeding (Rx); minor cuts, lacerations, and abrasions (OTC)	FDA 510(k) K133121, substantially equivalent determination in 2014	FDA submission included bench performance, ISO 10993 biocompatibility testing, and an *in vivo* swine hemostasis model; clinical testing was explicitly reported as not required for the submission	(U.S. Food and Drug Administration, 2014)
AnsCare ChitoClot Gauze	Nonwoven hemostatic dressing derived from chitosan fibers	Temporary external control of bleeding	FDA 510(k) K143462, cleared in 2015	The cited FDA submission establishes composition, performance, and substantial equivalence; no comparative human outcome study is presented in the cited regulatory evidence	(U.S. Food and Drug Administration, 2015a)
Axiostat family	Chitosan-based hemostatic dressing available in dressing, patch, and gauze configurations	Traumatic bleeding and wound/puncture-site hemostasis	FDA 510(k) records include K172324, K202830, and K222909	Prehospital randomized study, n = 104: mean hemostasis time 4.68 ± 1.04 min with Axiostat versus 18.56 ± 5.04 min with cotton gauze; successful hemostasis 94% versus 74%. Conversely, the prospective R3A transradial study was terminated after 41 patients following radial artery thrombosis in 19.5% (8/41), despite approximately 78% hemostatic success	(U.S. Food and Drug Administration, 2026b; Kabeer et al., 2019; Nakache et al., 2024)
InnoSEAL Hemostatic Pad	Lyophilized porous sponge containing chitosan and catechol-conjugated chitosan	Hemostasis at bleeding wounds and vascular puncture sites	FDA 510(k) K161013, cleared in 2016	Single-center RCT after transfemoral TACE: InnoSEAL n = 48 versus Clo-Sur PLUS P.A.D. n = 52. No major or moderate access-site complications occurred in either group; overall minor complications were 6.3% versus 19.2% (p = 0.075), and time to hemostasis did not differ significantly	(U.S. Food and Drug Administration, 2016; Seol et al., 2021)
S.E.A.L. Hemostatic Wound Spray	Chitosan dry powder delivered in spray form	Temporary control of severe topical bleeding (Rx); minor bleeding from cuts, lacerations, and abrasions (OTC)	FDA 510(k) K210751, substantially equivalent determination on 2 February 2023	Clearance was supported by *in vitro* and animal performance data rather than human clinical evidence. The FDA summary reported severe cell lysis/toxicity in an extract-based cytotoxicity assay and irritation with a sesame-oil extract; these findings were considered in the submission’s overall benefit–risk assessment for limited-duration use	(U.S. Food and Drug Administration, 2023a)
TRAUMAGEL/TRAUMAGEL 2.0	Chitosan granules suspended in a sodium-alginate gel and delivered from a prefilled syringe	Temporary external control of moderate-to-severe bleeding	TRAUMAGEL: FDA K240713, cleared 14 August 2024; TRAUMAGEL 2.0: K253609, cleared 12 December 2025	Regulatory evidence is based on substantial-equivalence and nonclinical testing. TRAUMAGEL 2.0 is indicated for external use with contact ≤24 h and is not intended for surgical use or wound closure	(U. S. Food and Drug Administration, 2026)
MFUSE	Polyacrylic acid in a plant-derived chitosan matrix, combined with mineral oil to form a syringe-delivered paste/hydrogel	Temporary external control of moderate-to-severe bleeding	FDA 510(k) K250319, cleared 15 December 2025	FDA submission reports biocompatibility and bench testing together with a GLP porcine carotid arteriotomy study demonstrating substantially equivalent performance to an FDA-cleared comparator; no human clinical outcome data were included in the submission	(U.S. Food and Drug Administration, 2025b)
KA01 Chitosan Wound Dressing	Sterile nonwoven dressing composed of 100% chitosan fibers; forms a gel after absorbing exudate	Moderately-to-heavily exuding acute and chronic wounds	FDA 510(k) K143124, cleared 26 January 2015	Multicenter randomized clinical study, n = 90: 4-week wound-area reduction 65.97% ± 4.48% with the chitosan dressing versus 39.95% ± 4.48% with conventional Vaseline gauze; pain scores were also lower	(U.S. Food and Drug Administration, 2015b; Mo et al., 2015)
SURECELL Gelling Fiber Wound Dressing	Hydrophilic nonwoven fiber composed of chitosan and chitosan derivatives; forms a gel in contact with exudate	Chronic, postoperative, traumatic, and selected minor wounds	FDA 510(k) K173005, cleared 25 May 2018	The cited FDA submission reports bench performance, biocompatibility, sterilization-validation, and shelf-life testing; comparative human efficacy data are not presented in the cited 510(k) evidence	(U.S. Food and Drug Administration, 2018)
MaxioCel Chitosan Wound Dressing	Soft, sterile, absorbent gelling chitosan wound dressing	Moderately-to-heavily exuding acute and chronic wounds	FDA 510(k) K212766, cleared 24 August 2023	An open-label multicenter randomized comparison with Aquacel Extra is registered as NCT05312762. No peer-reviewed outcome publication from this trial was identified during the present verification	(U.S. Food and Drug Administration, 2023b; ClinicalTrials.gov, 2026)
Foundation DRS Solo/Foundation DRS + Duo	DRS Solo: porous shellfish-derived chitosan–glycosaminoglycan scaffold; DRS + Duo additionally contains hyaluronic acid and a semipermeable polyurethane backing layer	Full- and partial-thickness wounds, ulcers, burns, donor sites, traumatic wounds, and surgical wounds	Foundation DRS Solo: FDA K210949 and K231937; Foundation DRS + Duo: K240298, cleared 21 August 2024	FDA documentation describes the porous scaffold as supporting cellular invasion and capillary growth. DRS + Duo was evaluated using biocompatibility, functional testing, and porcine wound-healing/implantation studies; clinical testing was not necessary to demonstrate substantial equivalence	(U.S. Food and Drug Administration, 2026c)
BST-CarGel	Chitosan-based scaffold mixed with autologous whole blood and applied in conjunction with microfracture	Focal articular-cartilage repair	Evaluated in an international multicenter randomized clinical program; no regulatory-market claim is made here	RCT, n = 80: greater lesion filling and repair-tissue T2 relaxation times closer to native cartilage at 12 months compared with microfracture alone. MRI-defined structural benefit persisted over 5 years; WOMAC scores improved substantially in both groups without a significant between-group difference	(Stanish et al., 2013; Shive et al., 2015)
Reaxon Plus/chitosan nerve-tube platform	Flexible, transparent, bioresorbable chitosan nerve tube/nerve cuff	Peripheral nerve discontinuities and adjunctive protection of primary nerve repair	Reaxon Plus: FDA Class II nerve cuff, K180222, decision 24 April 2018. A Reaxon Nerve Guide study comparing nerve-gap bridging with autologous grafting was registered as NCT02459015 and terminated before planned completion	The Reaxon nerve-gap study and the adjunctive-tube RCT should be distinguished. Separately, bicentric RCT n = 74 evaluated a chitosan tube around primary microsurgical coaptation and reported improved short-term sensory recovery; long-term follow-up identified increased pain and impaired sensory outcomes in selected tube groups and incomplete degradation at up to 5 years	(Neubrech et al., 2018; U.S. Food and Drug Administration, 2010; ClinicalTrials.gov, 2025; Aman et al., 2025)
Chitogel/Deferiprone–Chitogel	*In situ* chitosan–dextran hydrogel; investigational spinal formulation additionally contains deferiprone	Postoperative sinonasal wound management; investigational prevention of epidural fibrosis after lumbar surgery	Chitogel Endoscopic Sinus Surgery Kit: Australian ARTG ID 383512, Class III medical device	RCT n = 40 after endoscopic sinus surgery: mean hemostasis approximately 2 versus 10 min and significantly fewer adhesions with Chitogel. A subsequent randomized trial demonstrated reduced ostial stenosis. Later human studies evaluated Chitogel with therapeutic adjuvants. A single-arm Phase I Deferiprone–Chitogel study enrolled 20 lumbar-surgery patients and primarily established safety and feasibility; antifibrotic efficacy remains unconfirmed	(Therapeutic Goods Administration and Australia, 2022; Valentine et al., 2010; Vediappan et al., 2023; Candy et al., 2026; Ha et al., 2013)

Hemostasis represents the most extensively translated application of chitosan-based medical devices. HemCon Bandage and subsequent HemCon, ChitoFlex, and ChitoGauze configurations received multiple FDA 510(k) clearances, and clinical experience includes both military use and vascular-access hemostasis (Wedmore et al., 2006; U.S. Food and Drug Administration, 2020; Bachtell et al., 2006). In a randomized-order crossover study involving 50 hemodialysis patients, hemostasis within 4 min was achieved in 86% of HemCon-treated access sites compared with 72% treated with conventional gauze (Bachtell et al., 2006). Celox provides additional comparative human evidence: in a randomized trial of 160 patients with penetrating limb injuries, hemostasis within 5 min was achieved in 51.3% of patients treated with Celox-coated gauze compared with 32.5% treated with conventional gauze, with significantly lower blood loss in the Celox group (U.S. Food and Drug Administration, 2025a; Hatamabadi et al., 2015).

Clinical performance, however, cannot be generalized across products or indications. In a prehospital study of 104 patients with bleeding scalp wounds, Axiostat reduced mean time to hemostasis from 18.56 ± 5.04 min with conventional cotton gauze to 4.68 ± 1.04 min, with successful hemostasis in 94% and 74% of patients, respectively (Kabeer et al., 2019). By contrast, the prospective R3A study evaluating Axiostat for transradial access-site hemostasis was terminated prematurely after 41 patients because radial artery thrombosis occurred in 19.5% (8/41), despite successful hemostasis in approximately 78% of cases (Nakache et al., 2024). These contrasting findings illustrate an important translational principle: clinical performance belongs to a defined final device used under defined anatomical, procedural, and exposure conditions and should not be extrapolated from chitosan as a polymer class.

Wound-care products extend clinical translation beyond immediate bleeding control. KA01, a nonwoven dressing composed of 100% chitosan fibers, was evaluated in a multicenter randomized clinical trial involving 90 patients. After 4 weeks, wound-area reduction was greater with the chitosan dressing than with conventional Vaseline gauze (65.97% ± 4.48% versus 39.95% ± 4.48%), and pain scores were lower (U.S. Food and Drug Administration, 2015b; Mo et al., 2015). MaxioCel is an FDA-cleared chitosan wound dressing for which a randomized, open-label multicenter comparison with the carboxymethylcellulose dressing Aquacel Extra has been registered (NCT05312762); no peer-reviewed outcome publication from this trial was identified during the present verification (U.S. Food and Drug Administration, 2023b; ClinicalTrials.gov, 2026). In contrast, the cited FDA submissions for SURECELL and Foundation Dermal Regeneration Scaffold products rely predominantly on nonclinical performance, biocompatibility, sterilization, and related testing, with clinical testing not required to establish substantial equivalence for Foundation DRS + Duo (U.S. Food and Drug Administration, 2018; U.S. Food and Drug Administration, 2026c). These examples reinforce that regulatory clearance and the strength of direct human efficacy evidence represent distinct dimensions of clinical translation.

Cartilage repair provides comparatively robust randomized human evidence for a chitosan-based regenerative scaffold. In an international multicenter randomized controlled trial involving 80 patients, BST-CarGel, a chitosan-based scaffold mixed with autologous whole blood and applied in conjunction with microfracture, resulted in greater lesion filling and repair-tissue T2 relaxation times closer to native cartilage than microfracture alone at 12 months (Stanish et al., 2013). Longitudinal MRI analysis demonstrated that these structural differences persisted over 5 years. However, both groups showed substantial improvement in Western Ontario and McMaster Universities Osteoarthritis Index scores, without a significant between-group difference in patient-reported clinical outcomes (Shive et al., 2015). Thus, the available evidence supports a sustained MRI-defined structural advantage but does not establish long-term superiority in patient-reported clinical benefit.

Translation to peripheral nerve repair requires particular care in distinguishing between device clearance, nerve-gap bridging, and adjunctive use of chitosan tubes around primary nerve coaptation. Reaxon Plus is an FDA-cleared Class II chitosan nerve cuff indicated for repair of peripheral nerve discontinuities where gap closure can be achieved by flexion of the extremity (U.S. Food and Drug Administration, 2010). A registered clinical study comparing the Reaxon Nerve Guide with autologous nerve grafting for digital nerve defects (NCT02459015) was terminated before planned completion (ClinicalTrials.gov, 2025). Separately, a bicentric randomized controlled trial involving 74 patients evaluated an additional chitosan nerve tube around primary microsurgical repair of traumatic sensory nerve lesions and reported improved short-term sensory recovery (Neubrech et al., 2018). Long-term follow-up of this randomized cohort subsequently demonstrated increased pain and impaired sensory outcomes in selected tube groups, with persistent implant remnants detected by magnetic resonance neurography at up to 5 years (Aman et al., 2025). These findings demonstrate that an early regenerative advantage should not be assumed to translate into durable clinical benefit and highlight the importance of long-term degradation and tissue-response data for resorbable nerve-guidance materials.

Chitogel provides a further example of translation beyond conventional topical dressings. The Chitogel Endoscopic Sinus Surgery Kit is included in the Australian Register of Therapeutic Goods as a Class III medical device (Therapeutic Goods Administration and Australia, 2022). In a randomized controlled study involving 40 patients undergoing endoscopic sinus surgery, the chitosan–dextran gel reduced mean time to hemostasis from approximately 10 min in untreated control sites to 2 min and significantly reduced postoperative adhesion formation (Valentine et al., 2010). A subsequent blinded randomized trial demonstrated reduced postoperative ostial stenosis compared with untreated control sites (Ha et al., 2013). Further clinical investigation has evaluated Chitogel alone and in combination with therapeutic adjuvants following endoscopic sinus surgery (Vediappan et al., 2023). More recently, Deferiprone–Chitogel was evaluated in a single-arm Phase I pilot study involving 20 patients undergoing lumbar decompression surgery. This first-in-human spinal study primarily supported short-term safety and feasibility; because it lacked a concurrent control group and was not designed to establish antifibrotic efficacy, clinical efficacy remains to be determined in larger controlled trials (Candy et al., 2026).

Collectively, the clinical landscape remains markedly asymmetric. Translation is most advanced for systems in which the principal therapeutic function remains predominantly material-driven, including hemostasis, exudate management, wound coverage, and structural guidance. By contrast, growth-factor-, extracellular-vesicle-, cell-, and bioink-based chitosan systems discussed later in this review remain predominantly preclinical. As functional complexity increases, the translational bottleneck extends beyond polymer processing to include final-product identity, potency, release behavior, manufacturing reproducibility, sterilization, long-term material fate, and regulatory classification. Because regulatory clearances, trial status, and commercial availability may change over time, current status should be confirmed against the relevant regulatory or clinical-trial database.

## 2 Raw material - Source, parameters, and variability

Chitosan is derived from chitin, a biopolymer abundant in arthropod exoskeletons and some fungal cell walls. Industrially, it is obtained mainly from marine by-products, particularly shrimp and crab shells, which contain approximately 15%–30% chitin together with proteins and CaCO_3_. For medical-grade applications, however, marine by-products used as chitin sources require controlled sourcing, processing, and rigorous quality assessment to ensure consistency and safety (Hosney et al., 2022; Gîjiu et al., 2022; Younes and Rinaudo, 2015). Chitin is purified by demineralization (acid, typically HCl) and deproteinization (strong base such as NaOH) to minimize ash and protein content, and is subsequently deacetylated, converting N-acetyl-D-glucosamine units into D-glucosamine units to produce chitosan (Hosney et al., 2022; Zainol Abidin et al., 2020; Egorov et al., 2025). Crustacean sources offer high chitin content and mature extraction technology (Baharlouei and Rahman, 2022) but are seasonal and require removal of marine allergens (Varun et al., 2017). Fungal alternatives (e.g., *Mucor*, *Rhizopus*, and higher basidiomycetes), whose walls contain chitin covalently linked to β-glucan and partly deacetylated by endogenous deacetylases (Araújo et al., 2020; Davis and Bartnicki-García, 1984), allow year-round bioreactor cultivation free of mineral impurities and animal proteins, avoiding shellfish-allergen risk (Hussain et al., 2025; Fai et al., 2011); their drawbacks are lower chitin content (10%–20% dry weight), more complex separation from glucans, and limited yields at higher cost (Hussain et al., 2025; Seangarun et al., 2025; Amorim et al., 2001). The source of chitosan also influences its physicochemical properties, with crustacean-derived chitosan typically showing a higher MW, reaching up to several hundred kDa, but a lower DD, whereas fungal chitosan is usually more highly deacetylated, approximately 85%–90%, and has a lower MW, as illustrated by reported values of DD approximately 71% and MW approximately 354 kDa for crustacean chitosan versus DD approximately 85% and MW approximately 70 kDa for fungal chitosan (Hosney et al., 2022; Aberoumand and Chabavi, 2024; Hahn et al., 2020; Cheung et al., 2015).

### 2.1 Impact of deacetylation route on chitosan structure and variability

Deacetylation removes acetyl groups from N-acetylglucosamine units; once DD exceeds ∼50% the polymer becomes acid-soluble and is termed chitosan (Pochanavanich and Suntornsuk, 2002). Two routes are used: chemical and enzymatic (Egorov et al., 2025).

#### 2.1.1 Chemical route

Purified chitin is treated with 40%–50% NaOH at 100 °C–150 °C, often under pressure; alkaline hydrolysis cleaves amide bonds, while acidic media are avoided because they cleave glycosidic bonds (Egorov et al., 2025; Gonçalves et al., 2021). Temperature, reaction time, alkali concentration, and the number of treatment cycles control both DD and MW, as increasing any of these parameters generally increases DD while accelerating depolymerization and reducing MW, with repeated cycles leading to a particularly marked decrease in MW (Egorov et al., 2025; Tokatlı and Demirdöven, 2017; Hamdi et al., 2025). Deacetylation is non-uniform, creating acetyl-rich “hot spots” and a heterogeneous GlcNAc/GlcN distribution that can limit solubility despite high average DD, while chain scission broadens the MW distribution (Gonçalves et al., 2021; Aiba, , 1991; Kumar et al., 2004). An N_2_ atmosphere or NaBH_4_ can protect the backbone at added cost (Trombotto et al., 2008), and microwave-assisted processing shortens reaction times to minutes at comparable DD (Tokatlı and Demirdöven, 2017). Despite its environmental burden, the chemical route remains dominant for its simplicity and throughput (Egorov et al., 2025).

#### 2.1.2 Enzymatic route

Chitin deacetylases (CDA; EC 3.5.1.41) from fungi such as Mucorales catalyze N-deacetylation under mild aqueous conditions (∼40 °C–50 °C, neutral pH) that minimize glycosidic cleavage, giving higher MW, narrower distributions, more uniform structures, and DD tunable by enzyme dose and time (Stasińska-Jakubas and Hawrylak-Nowak, 2022; Ploydee and Chaiyanan, 2014; Hamdi et al., 2025). CDAs act processively, forming block-like deacetylated sequences that influence solubility and bioactivity (Araki and Ito, 1975). Efficiency is limited because enzymes attack only accessible crystal surfaces, requiring pretreatment (partial hydrolysis or milling); reported DD is often 60%–80% (occasionally 84%–90%) at modest yields, so research focuses on enzyme engineering and process optimization for biomedical-grade control (Stasińska-Jakubas and Hawrylak-Nowak, 2022; Kumirska et al., 2010; Hamdi et al., 2025). Source- and process-dependent variability and the two routes are summarized in Figure 3.

**FIGURE 3:**
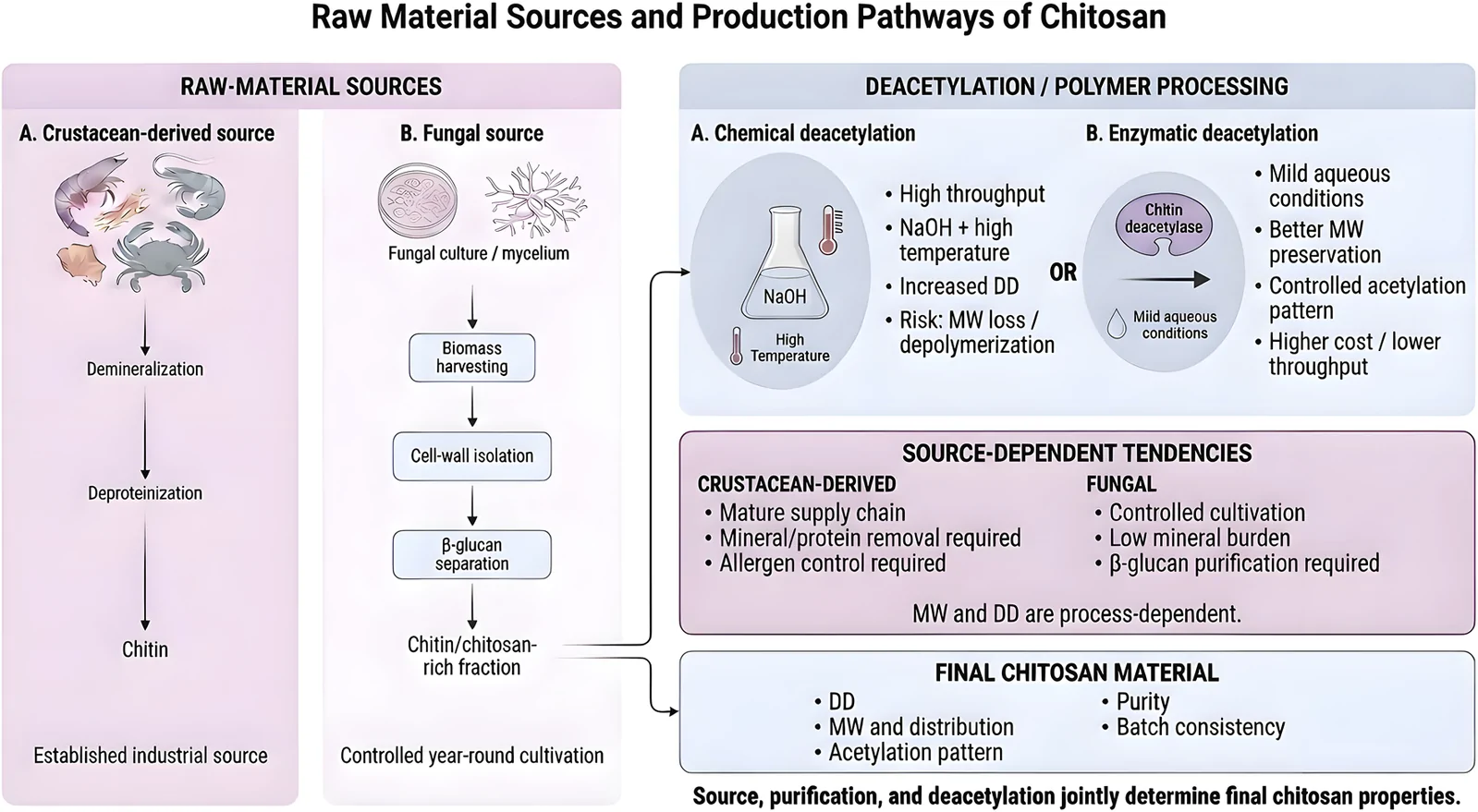
Source- and process-dependent variability of chitosan. Crustacean- and fungal-derived chitosans differ in mineral content, allergen-related concerns, yield, DD, MW, and purification requirements. Chemical and enzymatic deacetylation further shape depolymerization, acetylation pattern, scalability, and biomedical suitability. This figure was prepared with the assistance of FigureLabs.ai.

Importantly, the deacetylation route should therefore be regarded not only as a production step but also as a source of process-dependent variability in the final chitosan material. Differences in DD, MW, molecular-weight distribution, and acetylation pattern generated during processing may subsequently affect physicochemical properties, biological performance, and batch-to-batch reproducibility. Controlling and reporting these process-dependent attributes is therefore essential for the standardization of chitosan intended for biomedical applications.

### 2.2 Structural and physicochemical properties of chitosan

Chitosan is a linear β-1,4-linked copolymer of 2-amino-2-deoxy-D-glucopyranose (GlcN) and N-acetyl-D-glucopyranose (GlcNAc). It therefore represents a family of polymers that differ in the GlcN/GlcNAc ratio and chain length (Edo et al., 2024; Koirala et al., 2025; Gholap et al., 2024). The degree of deacetylation (DD), defined as the GlcN fraction, distinguishes chitosan from chitin, with chitosan generally defined by DD values above 50% (Younes and Rinaudo, 2015). A higher DD provides more protonatable -NH_2_ groups, which confer acid solubility, cationic character, and stronger antibacterial activity and tissue adhesion. In contrast, low-DD materials are poorly soluble and generally less bioactive (Edo et al., 2024; Koirala et al., 2025; Mawazi et al., 2024).

Commercial chitosan grades typically span DD values of 70%–95% (Edo et al., 2024) and MW values from 10 to >1,000 kDa (Hamdi et al., 2025). MW governs viscosity, mechanical strength, and biodegradation. Accordingly, high-MW chitosan forms more robust films and hydrogels, whereas low-MW chitosan and chitooligosaccharides are more soluble and permeable, but mechanically weaker and more rapidly degraded. The optimal MW is therefore application-dependent (Hamdi et al., 2025; Koirala et al., 2025; Wang and Roman, 2023).

Biomedical design must therefore balance bioactivity, biocompatibility, and controlled biodegradation: materials that persist too long can trigger chronic reactions, whereas overly rapid degradation may compromise structural integrity (Edo et al., 2024; Koirala et al., 2025). High-DD/high-MW chitosan may be advantageous in applications requiring greater structural stability, whereas lower-MW or partially acetylated forms may be preferable for drug delivery and soft-tissue applications requiring greater solubility and faster resorption (Hamdi et al., 2025; Wang and Roman, 2023).

A third determinant is the pattern of acetylation, together with polydispersity. Two samples with identical DD may differ in solubility and bioactivity because random pattern of acetylation disrupts crystallinity and promotes dissolution, whereas block-like sequences form hydrophobic domains that hinder dissolution. Chemical routes usually produce irregular, mosaic-like PA, while enzymatic routes generate more regular sequences. Specific acetylation motifs may also modulate biological responses, including immune-related effects (Hamdi et al., 2025; Aiba, 1991; von Seggern et al., 2025).

### 2.3 Solubility of chitosan and the influence of acid type

As a free base, chitosan is insoluble in neutral water because intermolecular hydrogen bonding stabilizes its crystalline structure; it dissolves only when its amino groups (pK_a_ ≈ 6.3–6.5) are protonated under acidic conditions, the resulting charge repulsion enabling solvation (Egorov et al., 2025; Edo et al., 2024). Typical solvents are dilute organic acids (acetic, formic, lactic, citric) or mild inorganic acids such as 0.1 M HCl, with acetic acid most common because chitosan acetates are biocompatible and easily neutralized (Egorov et al., 2025). Strong mineral acids can promote hydrolysis and leave residual acid or salt species that alter pH, osmolarity, and cytocompatibility and must be removed (Koirala et al., 2025). A milder alternative is CO_2_-assisted dissolution: compressed CO_2_ generates carbonic acid *in situ*, protonating -NH_2_ groups without persistent acidic residues to yield neutral, biocompatible solutions or hydrogels; this minimizes washing, enables direct tissue application with low irritation, and is validated as a bioink component (Molina-Ramírez et al., 2021; Banach-Kopeć et al., 2022; Verma and Reddy, 2024).

### 2.4 Chemical modifications of chitosan

Native chitosan’s poor neutral-pH solubility and limited functionality drive chemical modification at -NH_2_/-OH groups to improve solubility or add functions such as antimicrobial or conductive properties (Egorov et al., 2025; Pereira Parchen et al., 2025; Gholap et al., 2024). Quaternization yields permanently cationic quaternary ammonium salts such as N,N,N-trimethyl chitosan (TMC), water-soluble near neutral pH with stronger mucoadhesion and membrane permeability for mucosal drug/vaccine delivery, though high quaternization can raise cytotoxicity, especially at low MW (Pudžiuvelytė et al., 2022; Mawazi et al., 2024; Egorov et al., 2025). Carboxymethylation attaches -CH_2_-COOH to give amphoteric carboxymethyl chitosan that swells or partly dissolves at neutral-to-basic pH, used in hydrogels and drug carriers (Gholap et al., 2024; Hamdi et al., 2025). Hydrophobic modification or copolymerization with fatty acids or poly (ε-caprolactone) produces amphiphilic systems that self-assemble into micelles or nanoparticles for hydrophobic drugs, while O-alkylation broadens solvent solubility (Pereira Parchen et al., 2025; Koirala et al., 2025; Al-Jbour et al., 2019). These routes enable the tuning of solubility, functional-group availability, and mechanical properties (Egorov et al., 2025), but the introduction of foreign groups may reduce biocompatibility or increase cytotoxicity and immunogenicity. Because complete removal of residual reagents used during chemical modification, including iodomethane in methylation/quaternization and monochloroacetic acid in carboxymethylation, is essential but costly, many modified chitosans remain at the *in vitro* or preclinical stage (Pereira Parchen et al., 2025; Hamdi et al., 2025; Koirala et al., 2025). The major routes and their translational constraints are summarized in Figure 4.

**FIGURE 4:**
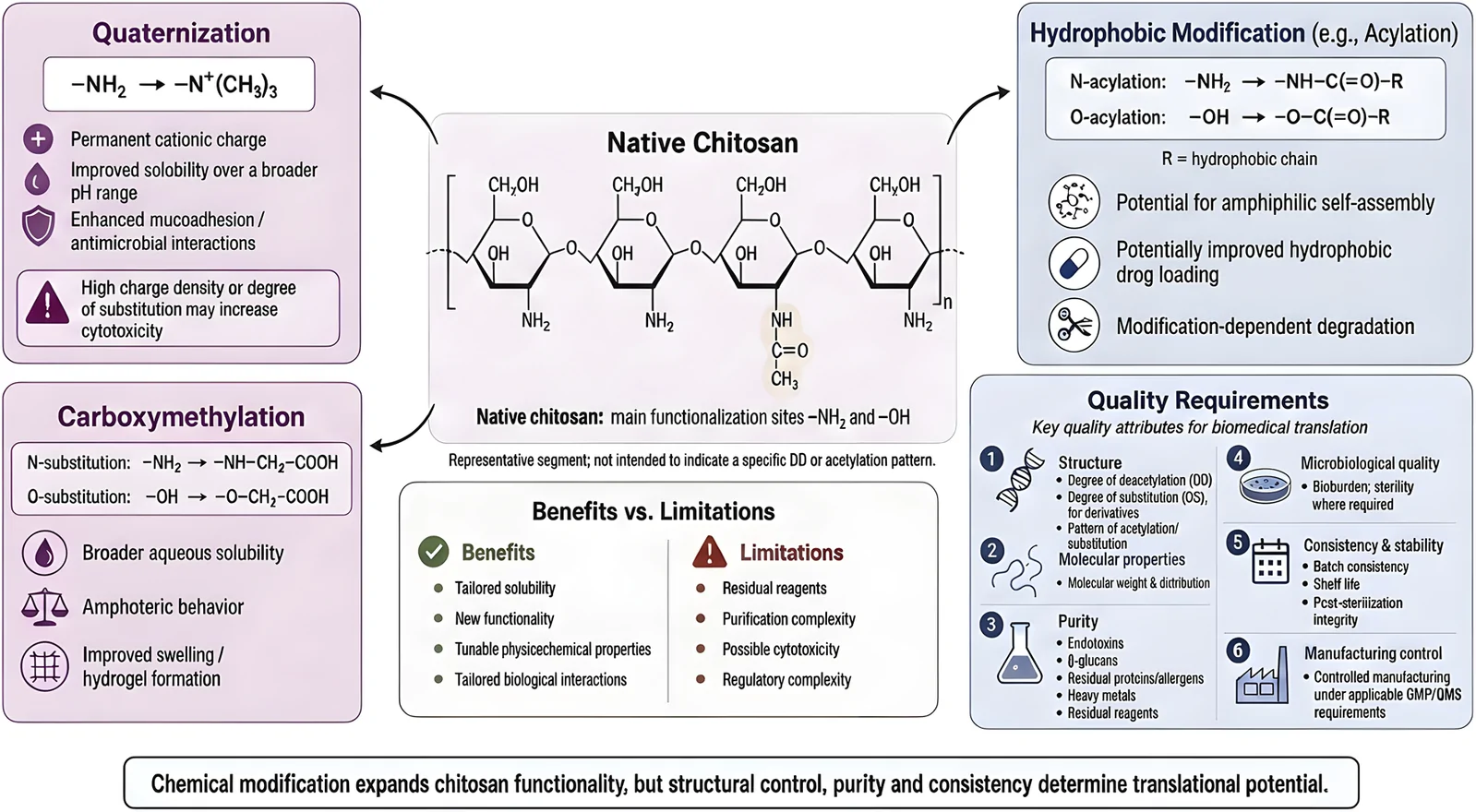
Chemical modification routes for tailoring chitosan functionality. Quaternization, carboxymethylation, and hydrophobic grafting can improve neutral-pH solubility, mucoadhesion, membrane interaction, self-assembly, and drug-loading capacity. Each modification also introduces translational constraints, including residual reagents, altered cytocompatibility, purification burden, and regulatory complexity. This figure was prepared with the assistance of FigureLabs.ai.

### 2.5 Purity, endotoxin control, and medical-grade quality requirements

Chitosan intended for biomedical use must meet stringent purity standards, since source- and process-derived impurities, including proteins, bacterial endotoxins, solvent or reagent residues such as NaOH and acids, heavy metals, pigments such as astaxanthin, other polysaccharides such as fungal β-glucan, and unreacted chitin, can affect biocompatibility, reproducibility, and safety (Ribeiro et al., 2009; Patrulea et al., 2015; Koirala et al., 2025).

#### 2.5.1 Proteins/allergens

Crustacean proteins such as shrimp tropomyosin are potent allergens, so chitosan should be essentially protein-free; modern extraction (repeated deproteinization, enzymatic proteolysis) reduces protein below 0.1%, and well-purified chitosan has shown no protein bands and no immunological events in seafood-sensitized individuals using chitosan dressings (Ribeiro et al., 2009; Waibel et al., 2011; Azad et al., 2004).

#### 2.5.2 Endotoxins

Lipopolysaccharide (LPS) cause strong pyrogenic responses, so permissible levels are very low (e.g., <0.1 EU/mg for implants) (Patrulea et al., 2015). Crustacean chitosan can acquire LPS during waste fermentation or poor sterility (Dai et al., 2011), and in fungal chitosan β-glucans interfere with the Limulus amebocyte lysate (LAL) test (factor G activation), making endotoxin-specific recombinant Factor C (rFC) preferable (Patrulea et al., 2015). Robust purification is achievable: iterative acid-dissolution/base-precipitation with exhaustive washing yielded endotoxin-free chitosan (Lebre et al., 2019); a chloroform-washed, pH 9-10 precipitated, autoclaved, CO_2_-redissolved protocol lowered LPS by ∼88–98% while preserving MW (Banach-Kopeć et al., 2022); and an optimized four-step NaOH protocol reduced endotoxin up to 100-fold while maintaining DD and MW (Reay et al., 2023).

#### 2.5.3 Mineral residues

Crustacean-derived chitosan may retain residual mineral salts, predominantly calcium compounds, when demineralization is incomplete. Ash content is therefore commonly used as an indicator of mineral contamination; values above 1% may indicate insufficient demineralization, whereas pharmacopoeial specifications generally require ash contents below 1%, with typical values of approximately 0.2%–0.5% (Ribeiro et al., 2009; Patrulea et al., 2015).

#### 2.5.4 Heavy metals

The concentrations of potentially toxic elements, particularly As, Cd, Hg, and Pb, must remain below accepted pharmacopoeial limits. Their control is particularly important for biomedical-grade chitosan because contamination may originate from the biological source material or from processing (Ribeiro et al., 2009; Patrulea et al., 2015).

#### 2.5.5 Moisture content

Chitosan is hygroscopic and typically contains approximately 8%–12% equilibrium moisture. Because water content can influence material mass, storage stability, and the reproducibility of physicochemical characterization, moisture should be controlled and reported as a quality parameter (Ribeiro et al., 2009; Patrulea et al., 2015).

#### 2.5.6 Quality requirements

Medical-grade chitosan must ensure consistent DD, appropriate MW, minimal impurities, and proven biocompatibility via standardized International Organization for Standardization (ISO)/FDA tests, with validated demineralization, deproteinization, decolorization, endotoxin reduction, reagent removal, and sterility/bioburden control confirmed per batch; for medical devices, only certified medical-grade material with a certificate of analysis, full traceability, and Good Manufacturing Practice (GMP)/ISO 13485 compliance should be used (Patrulea et al., 2015; Ribeiro et al., 2009; Koirala et al., 2025).

#### 2.5.7 Sterilization method and its effect on chitosan properties

For chitosan biomaterials intended to be supplied sterile, sterilization should be treated as a material-defining processing step rather than a neutral final operation, since its consequences depend on the method, physical state of chitosan, hydration, formulation composition, and processing dose (Table 2). Across the studies summarized in Table 3, no single method emerges as universally superior: γ-irradiation and electron-beam processing cause dose-dependent chain scission that scales with dose and physical state; steam autoclaving is compatible with some formulations but can substantially degrade chitosan solutions within minutes; ethylene oxide better preserves bulk structure in dry formats but is not chemically inert at the surface; and low-temperature approaches such as nitrogen plasma or supercritical-CO_2_ processing can avoid these trade-offs but introduce their own formulation-specific considerations. Sterilization method and dose should therefore be selected and validated for the final chitosan formulation, with post-sterilization testing focused on the attributes that determine its intended function.

**TABLE 2:** Functionalization strategies for chitosan-based regenerative biomaterials and critical translational controls.

Functionalization mode	Rationale	Critical controls	Representative evidence
Growth-factor adsorption or ionic binding	Simple loading and short-term local exposure	Burst release, displacement by serum proteins, release in relevant media, post-release bioactivity	Chitosan-based growth-factor systems can improve bone, wound, cartilage, and nerve models when dose and release are controlled (Chevrier et al., 2017; Obara et al., 2003; Li et al., 2017)
Affinity-based growth-factor retention	Heparin/chitosan or sulfated chitosan mimics extracellular matrix binding for heparin-binding factors	Sulfation pattern, anticoagulant activity, substitution degree, residual reagents, potency assays	2-N,6-O-sulfated chitosan modulated VEGF-induced angiogenesis; heparin/chitosan scaffolds retained NGF for peripheral nerve repair (Yu et al., 2018; Li et al., 2017)
Drug-loaded depots and staged delivery	Local antibiotic, anti-inflammatory, or osteogenic exposure matched to defect biology	Biofilm-relevant antimicrobial assays, host-cell toxicity, release after sterilization, resistance risk	Chitosan-containing scaffolds have been used for vancomycin or ciprofloxacin delivery and sequential antibiotic/BMP-2 release in infected bone contexts (Gao et al., 2023; Zhan et al., 2024; Zhang et al., 2024)
EV-loaded matrices	Local retention of source-defined extracellular vesicle fractions	Extracellular vesicle guideline-aligned source characterization, purity assessment, particle-to-protein metrics, evaluation of non-EV contaminants, release-normalized potency, and FBS controls	Chitosan hydrogels and nHA/CS/PLGA scaffolds have been reported to retain EV preparations and support wound or bone repair in preclinical models (Wu et al., 2024; Wan et al., 2024; Welsh et al., 2024)
Peptide immobilization	Stable presentation of integrin-binding or antimicrobial motifs	Ligand density, orientation, residual coupling agents, retained cell-binding activity	RGD and other ECM-derived peptides can improve cell attachment on chitosan films and scaffolds (Mochizuki et al., 2007; Brun et al., 2021; Forero et al., 2023)

**TABLE 3:** Experimentally demonstrated effects of sterilization approaches on chitosan-based biomaterials and their implications for translational material design. Reported outcomes are specific to the tested formulations and processing conditions and should not be interpreted as a universal ranking of sterilization methods.

Sterilization approach	Chitosan system and representative conditions	Experimentally observed effects	Translational implication	Primary evidence
Dry heat/saturated steam	Purified chitosan exposed to dry heat or saturated steam over a temperature range of 60 °C–160 °C and different exposure times	Heating altered the thermal behavior and solubility of chitosan. Increasing temperature and exposure severity reduced intrinsic viscosity and solubility and promoted discoloration, with saturated steam generally accelerating these changes	Thermal sterilization effects depend strongly on temperature, exposure time, and hydration. High-temperature or prolonged treatment may substantially modify the physicochemical properties of native chitosan	(Lim et al., 1999)
Steam autoclaving - thermogelling solution	2% (w/v) chitosan solution used in a chitosan/β-glycerophosphate thermogelling system	As little as 10 min of autoclaving caused approximately 30% reduction in molecular weight and a 3-5-fold decrease in dynamic viscosity, together with marked deterioration of gel mechanical properties; thermogelation at 37 °C remained possible	For injectable thermogelling systems, successful sterilization should be accompanied by post-sterilization assessment of rheological behavior and final gel mechanics	(Jarry et al., 2001)
Steam autoclaving - formulation-dependent response	Chitosan/β-glycerophosphate solutions containing 1%–5% polyols; separately, a chitosan-gluconic-acid conjugate solution autoclaved at 121 °C for 20 min	All tested polyols reduced autoclaving-induced viscosity loss, with PEG providing the greatest protection against hydrolytic degradation of chitosan chains (Jarry et al., 2002). In the chitosan-gluconic-acid system, autoclaving simultaneously induced hydrogel formation and sterilization while preserving properties compatible with the intended biological application (Yamashita et al., 2023)	Autoclaving is not intrinsically incompatible with chitosan hydrogels. Formulation composition can reduce thermal degradation or, in specifically designed systems, allow heat treatment to serve simultaneously as a sterilization and material-forming step	(Jarry et al., 2002; Yamashita et al., 2023)
γ-Irradiation - native chitosan	Purified, highly deacetylated chitosan fibers and films irradiated at doses up to 25 kGy	γ-Irradiation caused dose-dependent main-chain scission and reduction in viscosity-average molecular weight. Thermal properties were altered, whereas changes in tensile behavior did not directly parallel the reduction in molecular weight	Radiation dose alone is not sufficient to predict preservation of material performance; both molecular-level changes and application-relevant functional properties should be evaluated after irradiation	(Lim et al., 1998)
γ-Irradiation - dry powder	Dried chitosan powder subjected to γ-irradiation and compared with steam and ethylene oxide treatment	γ-Irradiation produced pronounced depolymerization of dry chitosan, with degradation increasing with irradiation dose	Drying does not eliminate radiation-induced degradation of chitosan chains; molecular-weight changes should therefore be assessed when γ-irradiation is applied to dry chitosan	(Yang et al., 2007)
γ-Irradiation - nanoparticle suspension	Aqueous chitosan-TPP hydrogel nanoparticles irradiated at 8, 13, and 25 kGy	Visible sedimentation occurred even at the lowest tested dose, accompanied by substantial changes in particle size, polydispersity index, and zeta potential. Addition of protective sugars increased resistance to irradiation-induced destabilization	Wet chitosan nanocarriers can be highly sensitive to irradiation; colloidal stability and particle characteristics should be reassessed after sterilization rather than inferred from results obtained with dry chitosan	(Gala et al., 2016)
γ-Irradiation - lyophilized drug-loaded nanoparticles	Lyophilized diclofenac-loaded N-trimethyl-chitosan nanoparticles irradiated at 5–25 kGy	The tested formulations retained their principal physicochemical characteristics over the investigated dose range. Initial bioburden was approximately 1.5 × 10^6^ CFU/vial, and approximately 9.9 kGy was calculated as sufficient to achieve the microbiological target defined in the study	Radiation tolerance is strongly formulation-dependent. The required sterilization dose should be established for the final product on the basis of its initial bioburden and post-irradiation quality attributes rather than extrapolated from unformulated chitosan	(Asasutjarit et al., 2017)
Ethylene oxide (EO) - dry powder	Dried chitosan powder sterilized with EO and compared with steam and γ-irradiation	EO caused comparatively limited changes in the assessed bulk structural characteristics of dry chitosan relative to steam treatment and γ-irradiation in the same study	EO may better preserve selected bulk properties of some dry chitosan materials, although this behavior should not be extrapolated to other formulations or to surface chemistry	(Yang et al., 2007)
Ethylene oxide - membranes	Chitosan, chitosan/glycerol, and chitosan/chitin membranes developed for wound-dressing applications	EO treatment did not significantly alter the evaluated membrane morphology or strain-at-break, whereas γ-irradiation adversely affected selected membrane properties	EO may be compatible with selected thin, dry chitosan-based constructs, but the response remains dependent on membrane composition and the specific functional property being assessed	(Marreco et al., 2004)
Ethylene oxide - surface chemistry	Chitosans with different degrees of deacetylation and carboxyethyl chitosan	Surface-sensitive analyses, including XPS and ToF-SIMS, demonstrated chemical modification of the chitosan surface after EO sterilization. The tested materials nevertheless showed acceptable *in vitro* cytocompatibility under the experimental conditions	Preservation of macroscopic morphology or bulk properties does not demonstrate chemical neutrality of EO sterilization. Surface chemistry should be evaluated when cell-material interactions are functionally important	(França et al., 2013)
Electron-beam irradiation	Solid alginate, chitosan, and alginate/chitosan polyelectrolyte-complex porous scaffolds irradiated at 2.5, 5, and 25 kGy using low-energy electron-beam processing	Electron-beam exposure produced dose- and material-dependent polymer degradation. Lower doses generally limited structural damage, while irradiation mode influenced the extent of polymer-chain degradation and preservation of porous scaffold architecture	Electron-beam conditions should be optimized for the specific polymer composition and scaffold architecture. Preservation of macroscopic morphology does not necessarily indicate preservation of molecular integrity or mechanical performance	(Farno et al., 2021)
Electron beam - lyophilized derivative	Lyophilized carboxymethyl chitosan irradiated at 15 and 25 kGy	The principal chemical structure of carboxymethyl chitosan was retained after irradiation, while dose-dependent changes in reconstituted-solution viscosity were observed. The irradiated material retained its ability to form stable hydrogels with suitable mechanical properties	Compatibility with electron-beam sterilization depends on derivative chemistry and physical state; results obtained for aqueous chitosan systems should therefore not be directly extrapolated to lyophilized derivatives	(Yuan et al., 2024)
Ozone	Aqueous chitosan-TPP hydrogel nanoparticles subjected to 2–10 ozone pulses	Ozonation caused comparatively limited changes in particle size and morphology, although polydispersity increased and zeta potential decreased with increasing treatment. Antimicrobial efficacy and biological response depended on treatment conditions and formulation composition; protective sugars reduced adverse biological effects, although chemical alterations remained detectable	Ozone may be considered for selected thermosensitive chitosan nanomaterials, but sterilization efficacy, formulation composition, gas exposure, chemical integrity, and biological response require product-specific validation	(Gala et al., 2016)
Non-thermal nitrogen plasma	Chitosan flakes and micronized dry powder treated using low-pressure or atmospheric-pressure nitrogen plasma	Sterile material was obtained. Endotoxin levels were 2.46 EU/g after low-pressure plasma treatment and 1.26 EU/g after atmospheric-pressure treatment. Reduced viscosity decreased following plasma exposure, particularly for micronized powder	Plasma treatment is a promising low-temperature approach for dry chitosan, but effects introduced during upstream processing must be distinguished from sterilization-induced changes because cryomilling itself altered molecular weight and degree of deacetylation	(Crofton et al., 2016)
Supercritical CO_2_ (scCO_2_)	Chitosan-TPP/PVA aerogels processed using integrated solvent exchange, scCO_2_ drying, sterilization, and naringin loading	The integrated process yielded sterile aerogels. The selected CS-TPP formulation retained high specific surface area (190.8 ± 9.8 m^2^/g), porosity (71.1% ± 1.4%), mechanical resistance, and low cytotoxicity under the tested conditions	scCO_2_ processing is particularly relevant to porous, drug-loaded chitosan constructs because sterilization can be integrated with drying and manufacturing. However, the demonstrated performance remains specific to the tested formulations and processing conditions	(Bento et al., 2025)

### 2.6 Mechanisms of chitosan interaction with cells and host tissues

#### 2.6.1 Physicochemical determinants of chitosan bioactivity

Chitosan’s biological behavior is set by MW, DD, acetylation pattern, polydispersity, purity, residual impurities, endotoxin content, and formulation format; materials sharing an average DD can still differ in charge density, crystallinity, solubility at physiological pH, lysozyme susceptibility, and protein interactions, producing divergent cellular responses even in the same cell line (C.-Eulalio et al., 2020; Younes and Rinaudo, 2015). DD determines the fraction of protonatable amino groups, whereas the distribution of acetylated residues, rather than the average DD alone, governs lysozyme-mediated cleavage, degradation rate, and persistence at implant sites (Younes and Rinaudo, 2015; Roman et al., 2020). MW shapes viscosity, diffusion, particle formation, and the dominant mode of action of chitosan, ranging from solution-based effects for low-MW polymers to surface- or scaffold-based functions for high-MW polymers (C.-Eulalio et al., 2020; Ravindranathan et al., 2016). Because chitosan is only partially protonated at physiological pH, its interactions are highly environment-dependent. Acidic exudate, endosomal compartments, and inflammatory microenvironments can increase electrostatic binding, whereas neutral pH and physiological salt concentrations screen charge and shift the dominant effects toward surface-mediated behavior. Claims based on acidic media, dilute solutions, or serum-free buffers should therefore not be directly extrapolated to blood-contacting or implanted systems. Crosslinking adds complexity, as adverse responses attributed to “chitosan” may stem from residual aldehydes, photoinitiators, unreacted methacrylate, acidic solvents, or extractables; mechanistic studies should therefore report chitosan chemistry, crosslinking, residual-crosslinker control, extractables, sterilization, and post-sterilization changes (Muzzarelli, 2009; Szymańska and Winnicka, 2015). Purity is equally decisive: even medium-MW chitosan can carry enough endotoxin to induce tumor necrosis factor (TNF-α) in peripheral blood mononuclear cell (PBMC) co-culture, and fungal or plant-derived inputs add β-glucan that interferes with LAL assays unless glucan-blocking or rFC methods are used, so credible immunological claims require endotoxin quantification, removal, spike-recovery, TLR4 blockade, and orthogonal testing (Reay et al., 2023; Banach-Kopeć et al., 2022; Patrulea et al., 2015; European Directorate for the Quality of Medicines and HealthCare, 2023; United States Pharmacopeia, 2024; Hussain et al., 2025).

#### 2.6.2 Protein adsorption and biomolecular corona formation

Most chitosan biomaterials do not contact cells as pristine polymers. Upon exposure to biological fluids, including plasma, serum, exudate, synovial fluid, saliva, mucus, or interstitial fluid, their surfaces rapidly adsorb proteins, glycoproteins, lipids, salts, and ECM components. Cells therefore interact with a dynamic biointerface, commonly termed a protein corona for particles, but conceptually relevant also to films, fibers, gels, and scaffolds (Ezzat et al., 2022). Although the cationic character of chitosan favors the adsorption of anionic proteins, corona composition is shaped by MW, DD, hydrophobic acetylated domains, surface texture, porosity, swelling, pH, ionic strength, protein concentration, and competitive adsorption. This adsorbed layer defines the effective ligand landscape presented to cells, which may respond to albumin, fibrinogen, immunoglobulins, complement fragments, vitronectin, fibronectin, or apolipoproteins rather than to chitosan itself (Ezzat et al., 2022). Folate-chitosan nanoparticles showed serum-source-dependent coronas that altered targeting, and hyaluronic-acid coating reduced corona-associated immunogenicity, indicating that many “chitosan” responses are interfacial properties of the formulated material (Ezzat et al., 2022; Almalik et al., 2017). Complement effects are especially relevant for injectable or blood-contacting formats, where opsonization can proceed through corona-associated proteins rather than recognition of the polymer itself (Marchand et al., 2010). The practical implication is that chitosan should not be tested only in serum-free conditions unless the clinical use is also protein-free: experiments should distinguish unconditioned, serum/plasma-conditioned, and ECM-coated chitosan, using corona proteomics or controlled serum comparisons for particles and protein pre-coating for scaffolds to separate polymer effects from protein-mediated ones (Ezzat et al., 2022; Almalik et al., 2017).

#### 2.6.3 Mucoadhesion, epithelial interactions, and membrane-level effects

Mucoadhesion is among the best-supported chitosan biointerface phenomena: protonated amines bind anionic mucin residues (sialic acid- and sulfate-bearing glycans), with hydrogen bonding, chain interpenetration, hydration, and hydrophobic domains as secondary contributors (Schipper et al., 1997). It should not be overgeneralized, because mucins differ by anatomical site, glycosylation, hydration, microbial environment, and disease, and binding measured with purified, gastric, porcine, or synthetic mucin may not predict behavior at human nasal, ocular, oral, intestinal, vaginal, or wound surfaces; MW, DD, crosslinking, and particle formation further shift residence time and convert soluble adhesion into particle or gel retention (Schipper et al., 1997). Chitosan-mediated, reversible opening of epithelial tight junctions is mechanistically supported and linked to defined intracellular pathways (Schipper et al., 1997; Zhang et al., 2014). However, this effect is context-limited, requiring soluble or partially soluble cationic chitosan, suitable pH, limited ionic screening, and accessible epithelium. Barrier-opening data from nasal or intestinal delivery should therefore not be extrapolated to dermal, bone, cartilage, or intramuscular implants.

Membrane-level effects are also formulation-dependent. Cationic chitosan can perturb membranes, glycosaminoglycans, and vesicle surfaces. In nanoparticles, positive charge can promote uptake, but also hemolysis, complement opsonization, and clearance, whereas in hydrogels or films the same charge may be buried or neutralized. Membrane interactions should therefore be reported together with zeta potential measured in the actual biological medium, rather than in water. The defensible summary is that chitosan can prolong residence at wet or mucus-rich interfaces and transiently modulate epithelial barriers under specific conditions, properties most relevant to topical, ocular, oral-mucosal, periodontal, and nasal delivery rather than to mineralized-tissue implants.

#### 2.6.4 Blood and host-tissue interactions

Hemostasis is one of the most clinically visible properties of chitosan. It arises from several concurrent mechanisms, including electrostatic erythrocyte aggregation, platelet adhesion and activation, fluid absorption, concentration of clotting components, tissue adhesion, and plug formation. Therefore, hemostatic efficacy depends not only on polymer chemistry, but also on porosity, fiber size, swelling capacity, and contact mechanics. It should not be attributed to chitosan alone (Gheorghiță et al., 2023; Wang et al., 2019).

Blood compatibility is not a single endpoint. A dressing intended for external hemorrhage may benefit from rapid blood cell aggregation, whereas an intravascular coating or injectable particle must avoid thrombosis, complement and platelet activation, and embolism. Thus, the same cationic interactions that are useful in topical hemostatic materials may be unacceptable in intravascular applications. Blood interactions should therefore always be interpreted in relation to the intended contact type, duration, anatomical site, and regulatory category.

The role of complement remains debated. Early studies reported alternative-pathway activation of C3 and C5, but not C4, by chitin and chitosan (Minami et al., 1998). Later studies in human plasma and serum found binding of C3, C5, and factor B without direct activation, and attributed C5a generation in coagulating whole blood to platelet and thrombin activity rather than direct complement triggering (Marchand et al., 2010). Other work indicates that the physicochemical state of chitosan, including solubility and acetylation homogeneity, can influence these outcomes (Guo et al., 2018). A conservative interpretation is that chitosan can bind complement proteins and participate in complement-coagulation cross-talk under specific conditions, but the general statement that chitosan activates complement is too broad. Complement should therefore be assessed using pathway- and activation-specific markers rather than inferred from protein binding alone (Marchand et al., 2010; Minami et al., 1998; Guo et al., 2018).

Inflammatory responses are similarly context-dependent. Acute cell recruitment may be inseparable from hemostasis in a dressing, but the same response may promote fibrosis and capsule formation around an implant. Degradation products, adsorbed proteins, residual acetic acid, crosslinker residues, endotoxin, β-glucans, and sterilization-induced changes can all distort cytokine readouts. For this reason, single-time-point inflammatory assays are insufficient to characterize the biological response to chitosan-based materials.

#### 2.6.5 Innate immune response

A key *in vivo* study showed that porous chitosan scaffolds with different degrees of acetylation induced distinct macrophage polarization profiles, linking a specific polymer parameter to host response and providing one of the stronger mechanistic anchors for chitosan immunobiology (Vasconcelos et al., 2013). However, this relationship should not be interpreted as a response to polymer chemistry in isolation. At the biomaterial interface, macrophages encounter not only the chitosan itself but also a complex interfacial environment comprising adsorbed proteins, material-derived debris, degradation products, and local injury-associated signals.

Nevertheless, polarization claims require caution. Many studies rely on limited M1/M2 marker panels, short culture periods, non-human cell lines, non-physiological media, and poorly characterized chitosan batches. Moreover, the binary M1/M2 framework oversimplifies biomaterial-induced inflammation, where macrophage phenotype is shaped by phagocytosis, hypoxia, stiffness, debris, and cytokine gradients. Therefore, an M1 or M2 claim is credible only when MW, DD, acetylation, endotoxin content, β-glucan contamination, sterilization, architecture, and temporal phenotype are described (Sadtler et al., 2016; Reay et al., 2023; Vasconcelos et al., 2013).

Endotoxin is a major confounder. Native chitosan can induce TLR4-dependent TNF-α production that disappears after purification, which undermines broad receptor-level claims when endotoxin is not controlled. Similarly, reported TLR4 signaling by chitosan oligosaccharides should be interpreted cautiously, since trace endotoxin can reproduce the same effect (Reay et al., 2023; Ravindranathan et al., 2016).

More specific mechanisms have also been reported. Chitosan can activate inflammasome pathways under endotoxin-minimized conditions, suggesting some degree of intrinsic innate immune activation. However, this response depends on particle size, cellular uptake, lysosomal stress, solubility, acetylation, and dose. Soluble oligomers, nanoparticles, and porous scaffolds should therefore not be assumed to share a single mechanism (Bueter et al., 2014).

Neutrophil interactions are less well resolved, but are likely relevant to wound dressings and infected defects. They remain under-quantified, particularly with respect to NET formation, neutrophil phenotype, myeloperoxidase activity, and neutrophil-to-macrophage transition.

Finally, the foreign-body reaction is not intrinsic to the polymer alone. Implant size, shape, pore size, mechanical mismatch, roughness, degradation rate, and vascular access all influence giant-cell formation, fibrosis, and vascularization. Chitosan-specific conclusions are therefore weak unless geometry and architecture are controlled (Abaricia et al., 2021).

#### 2.6.6 Cell-specific interactions relevant to regeneration

##### 2.6.6.1 Fibroblasts and keratinocytes

Fibroblast and keratinocyte responses are widely cited in the context of wound healing, but they usually reflect the behavior of the whole formulation rather than chitosan alone. Relevant formulation variables include hydration, stiffness, adhesive peptides, gelatin or collagen blending, residual acid, pore architecture, antimicrobial additives, and growth-factor loading. For example, peptide-modified chitosan hydrogels can accelerate repair, but such findings primarily demonstrate the value of added integrin-binding cues rather than intrinsic pro-regenerative activity of chitosan itself (Brun et al., 2021). Keratinocyte responses are also tissue- and formulation-specific. Chitosan-based systems may support re-epithelialization by maintaining moisture, reducing microbial burden, limiting fluid loss, or delivering bioactive factors, and some models show signaling changes, such as ERK activation in corneal epithelium. However, such results should not be directly transferred across epithelial tissues (Ribeiro et al., 2009). To isolate a polymer-intrinsic effect, studies should compare chitosan-based systems with matched non-chitosan dressings of similar moisture content, porosity, stiffness, degradation profile, and antimicrobial performance, a level of control that few studies achieve.

##### 2.6.6.2 Endothelial cells and angiogenesis

The general statement that chitosan is pro-angiogenic is not defensible. Chitosan scaffolds can support endothelial adhesion and tube formation when stiffness, porosity, hydration, and protein adsorption are appropriate. However, angiogenic outcomes may also arise from VEGF retention, heparin-mimetic functionalization, wound hypoxia, inflammation, or pore architecture (Yu et al., 2018). Some derivatives and biological contexts are instead associated with anti-angiogenic effects. For example, carboxymethyl and other modified chitosans have been studied in settings where angiogenesis suppression is desirable, indicating that the direction of the response depends on derivative chemistry and biological context (Yu et al., 2018). The strongest evidence comes from systems with an explicit mechanism, such as sulfated chitosan acting as a heparin-mimetic regulator of VEGF signaling. In such cases, the active material is a chemically modified glycosaminoglycan-mimetic polymer with growth-factor-binding properties. This is more mechanistically rational than attributing angiogenic effects to unmodified chitosan, but it also requires more detailed safety, reproducibility, and manufacturing characterization (Yu et al., 2018).

##### 2.6.6.3 Osteoblasts, chondrocytes, and MSCs

Osteoblast, chondrocyte, and mesenchymal stromal/stem cell (MSC) studies dominate the regenerative chitosan literature, particularly in bone and cartilage applications. However, these cells respond to many simultaneous cues, including adsorbed serum proteins, calcium phosphate or hydroxyapatite fillers, stiffness, pore architecture, degradation products, ionic composition, growth factors, and induction media. Therefore, lineage outcomes are often driven by the composite scaffold and culture medium rather than by chitosan alone (Chevrier et al., 2017). DD has been linked to protein adsorption and osteogenic response; however, a higher DD is often accompanied by a lower MW as a consequence of polymer-chain degradation during deacetylation. MW should therefore be considered as a potential confounding parameter when interpreting DD-dependent osteogenic responses. Consequently, the statement that higher DD promotes osteogenesis should be avoided unless MW, crystallinity, roughness, and protein adsorption are considered. In composite bone scaffolds, the mineral phase often provides the dominant osteoconductive cue, while chitosan contributes processability, porosity, drug binding, or degradation control (Chevrier et al., 2017; C.-Eulalio et al., 2020).

In cartilage applications, chitosan is typically combined with collagen, gelatin, hyaluronic acid, methacrylated networks, kartogenin, growth factors, or extracellular vesicles. In these systems, stiffness, swelling, oxygen tension, transforming growth factor (TGF-β) signaling, mechanical loading, and inflammatory cytokines are often decisive. Such formulations should therefore be interpreted as composite delivery or instructive platforms rather than chitosan-only chondrogenic materials (Liang et al., 2025). MSC studies require additional caution because MSCs are donor-dependent and heterogeneous. Chitosan-associated changes in adhesion, survival, paracrine secretion, or differentiation markers may not predict *in vivo* potency. Translational products should therefore be evaluated as combined scaffold-cell constructs under clinically relevant conditions rather than inferred from cell behavior on plastic (International Society for Stem Cell Research, 2021).

#### 2.6.7 Degradation and bioactivity of degradation products

Chitosan degrades in mammalian tissues mainly through lysozyme and related enzymes, including N-acetyl-β-D-glucosaminidase. However, degradation rate and degradation products depend on MW, DD, acetylation pattern, crystallinity, hydration, crosslinking, and the accessibility of acetylated sequences. Degradation is therefore enzymatic, interfacial, and tissue-dependent, rather than predictable from chemical structure alone, as is often assumed for ester-based synthetic polymers (Patrulea et al., 2015; Kumirska et al., 2010; Roman et al., 2020).

The acetylation pattern is particularly important because lysozyme requires suitable acetylated sequences for cleavage. As a result, two chitosans with similar average DD may degrade at different rates. Crosslinking, mineral phases, or dense scaffold architecture can further limit enzyme access, meaning that mass loss measured only in phosphate-buffered saline (PBS) is insufficient to predict *in vivo* residence time (Younes and Rinaudo, 2015; Roman et al., 2020).

Degradation products may be bioactive, but this claim requires specification. Chitosan oligosaccharides can interact with immune, epithelial, and microbial systems, but their effects depend on chain length, acetylation, purity, endotoxin contamination, and dose. Moreover, local oligosaccharide concentrations generated by a slowly degrading scaffold may be far lower than those used in soluble-oligomer experiments, so these two settings should not be treated as equivalent (Ravindranathan et al., 2016).

Practically, degradation should be matched to the intended regenerative window. Short-term degradation may be suitable for removable hemostatic dressings, whereas early bone repair may require temporary mechanical stability. Degradation-coupled behavior may be useful for growth-factor or extracellular vesicle (EVs) release, while cartilage applications may require longer residence. Accordingly, degradation should be tested in enzyme-containing, protein-rich, and mechanically relevant media rather than in simple buffer alone (Patrulea et al., 2015; Szymańska and Winnicka, 2015; Roman et al., 2020).

#### 2.6.8 *In vitro*, in vivo studies, and clinical observations

*In vitro* studies are useful for mechanistic analysis, but they often overpredict regenerative performance. Serum-free assays, non-physiological pH, supra-clinical polymer concentrations, uncontrolled endotoxin, absence of competing proteins, short time points, and immortalized cell lines can exaggerate cationic membrane effects, uptake, cytokine induction, or adhesion.

*In vivo*, tissue-level complexity can substantially change these responses. Bleeding, clot formation, protein adsorption, degradation, microbial burden, immune infiltration, vascularization, mechanical loading, and surgical injury may reverse *in vitro* impressions. A material that appears pro-inflammatory in macrophage culture may support repair by accelerating hemostasis and reducing contamination, whereas a material that appears cytocompatible may induce fibrosis if its architecture or degradation profile is inappropriate. Interpretation should therefore focus on the trajectory of the tissue response and the final tissue outcome rather than on single mechanistic labels.

Clinical evidence is strongest for topical wound and hemostatic applications, but remains device- and indication-specific. For example, a multicenter RCT comparing a chitosan-derivative hydrogel paste with a commercial hydroactive gel showed granulation improvement in both groups without clear superiority, indicating that plausible preclinical biology does not necessarily translate into superior clinical outcomes (Mohd Nasir et al., 2022).

A credible chitosan mechanism should therefore be stated conditionally. A defined chemistry, in a defined format, under defined exposure conditions, produces a defined biointerface and tissue response. This framing replaces universal labels such as “biocompatible” or “pro-regenerative” with parameterized and testable claims. The principal chitosan-host mechanisms, their key material determinants, representative evidence, and translational implications are summarized in Table 4.

**TABLE 4:** Mechanistic evidence and translational implications for chitosan-host biointerface responses.

Mechanism	Key material determinants	Representative evidence	Translational implication
Protein adsorption and corona formation	DD, MW, surface charge, hydrophobic acetylated domains, serum source, particle/surface architecture	Serum source changed the protein corona and targeting of folate-chitosan nanoparticles; hyaluronic-acid coating reduced corona-associated immunogenicity (Ezzat et al., 2022; Almalik et al., 2017)	Test formulated materials in protein-containing media and distinguish polymer effects from protein-conditioned biointerfaces
Mucoadhesion and epithelial interaction	Protonation state, pH, ionic strength, mucin type, solubility, nanoparticle versus gel format	Cationic chitosans enhance mucosal residence and can transiently modulate epithelial tight junctions under defined delivery conditions (Schipper et al., 1997; Zhang et al., 2014)	Do not extrapolate barrier-opening data from mucosal delivery to mineralized-tissue scaffolds without application-specific evidence
Hemostasis and blood contact	Surface accessibility of amines, porosity, absorption, dressing architecture, blood-contact duration	Chitosan dressings support erythrocyte and platelet interactions, whereas complement activation data differ by assay and material state (Wang et al., 2019; Minami et al., 1998; Marchand et al., 2010)	Topical hemostatic benefit does not imply intravascular hemocompatibility; hemolysis, coagulation, platelet, and complement assays should be product-specific
Innate immune response	Acetylation, endotoxin, β-glucan, particle size, degradation, architecture, sterilization	Degree of acetylation altered macrophage polarization in implanted porous scaffolds, and purified chitosan can activate NLRP3 inflammasome pathways under controlled conditions (Vasconcelos et al., 2013; Bueter et al., 2014)	Endotoxin and β-glucan controls are essential before assigning cytokine or macrophage phenotypes to chitosan itself
Degradation and remodeling	MW, DD, pattern of acetylation, crystallinity, hydration, crosslinking, enzyme exposure	Lysozyme-mediated degradation depends on acetylation chemistry and molecular architecture rather than average DD alone (Roman et al., 2020; Kumirska et al., 2010)	Degradation testing should use enzyme- and protein-containing media that approximate the target tissue and regenerative time window

### 2.7 Antimicrobial properties: Soluble polymer versus final biomaterial

Chitosan is widely studied as an antimicrobial agent. Its cationic amino groups interact electrostatically with anionic microbial membranes, disrupting membrane integrity and potentially causing cell lysis. This activity is strongly influenced by DD and MW (Rabea et al., 2003; Raafat and Sahl, 2009; Kong et al., 2010; Dhlamini et al., 2024).

However, antimicrobial activity is not an intrinsic and universal property of every chitosan-containing material. It depends on the physicochemical state of the polymer, including whether chitosan is soluble and protonated, incorporated into a biomaterial, crosslinked, or immobilized on a surface (Raafat and Sahl, 2009; Kong et al., 2010; Ke et al., 2021). Higher DD increases amino-group density and may therefore enhance antimicrobial activity (Dhlamini et al., 2024). Nevertheless, many chitosan hydrogels and xerogels show reduced or absent activity compared with pure chitosan. This is because soluble chitosan, particularly under acidic conditions that promote protonation, can directly contact microbial cells, whereas formulated systems may restrict chain mobility, reduce amino-group accessibility, or alter the local pH and ionic environment (Egorov et al., 2025; Rabea et al., 2003; Raafat and Sahl, 2009; Kong et al., 2010; Ke et al., 2021).

Formulation effects further complicate this relationship. Additives may shield positive charge, modify swelling, or limit direct microbial contact, meaning that the mere presence of chitosan does not guarantee retained antimicrobial activity (Dhlamini et al., 2024; Ke et al., 2021; Farasati Far et al., 2024; Ardean et al., 2021). MW is another important variable. Lower-MW chitosan is more active in some systems, whereas higher-MW chitosan is more effective in others. Reported MIC values therefore span several orders of magnitude, and effective concentrations may exceed clinically feasible doses in some settings. Physiological pH and ionic strength can also reduce protonation and activity, while low chitosan content or suboptimal DD may weaken efficacy (Raafat and Sahl, 2009; Kong et al., 2010; Dhlamini et al., 2024; Ke et al., 2021; Teixeira-Santos et al., 2021; Ciarlantini et al., 2024).

Crosslinked or surface-bound systems should be evaluated separately. In these formats, antimicrobial performance depends on surface charge density, wettability, swelling, roughness, amino-group accessibility, and direct microbial contact. Strong crosslinking may reduce activity by consuming amino groups or restricting polymer mobility (Ke et al., 2021; Ardean et al., 2021; Teixeira-Santos et al., 2021).

Because microbial susceptibility also varies by organism, antimicrobial studies should report chitosan source, DD, MW, concentration, solubility state, solvent, final pH, ionic strength, strain, inoculum, exposure time, method, formulation, crosslinking, sterilization, and whether chitosan is soluble, entrapped, crosslinked, or surface-bound (Egorov et al., 2025; Raafat and Sahl, 2009; Kong et al., 2010; Dhlamini et al., 2024; Ke et al., 2021). This level of reporting is necessary to distinguish genuine antimicrobial activity from formulation- or assay-dependent artifacts. Chitosan should therefore be regarded as a condition-dependent antimicrobial biomaterial whose performance must be validated for each final formulation rather than assumed from composition alone (Figure 5) (Egorov et al., 2025; Dhlamini et al., 2024).

**FIGURE 5:**
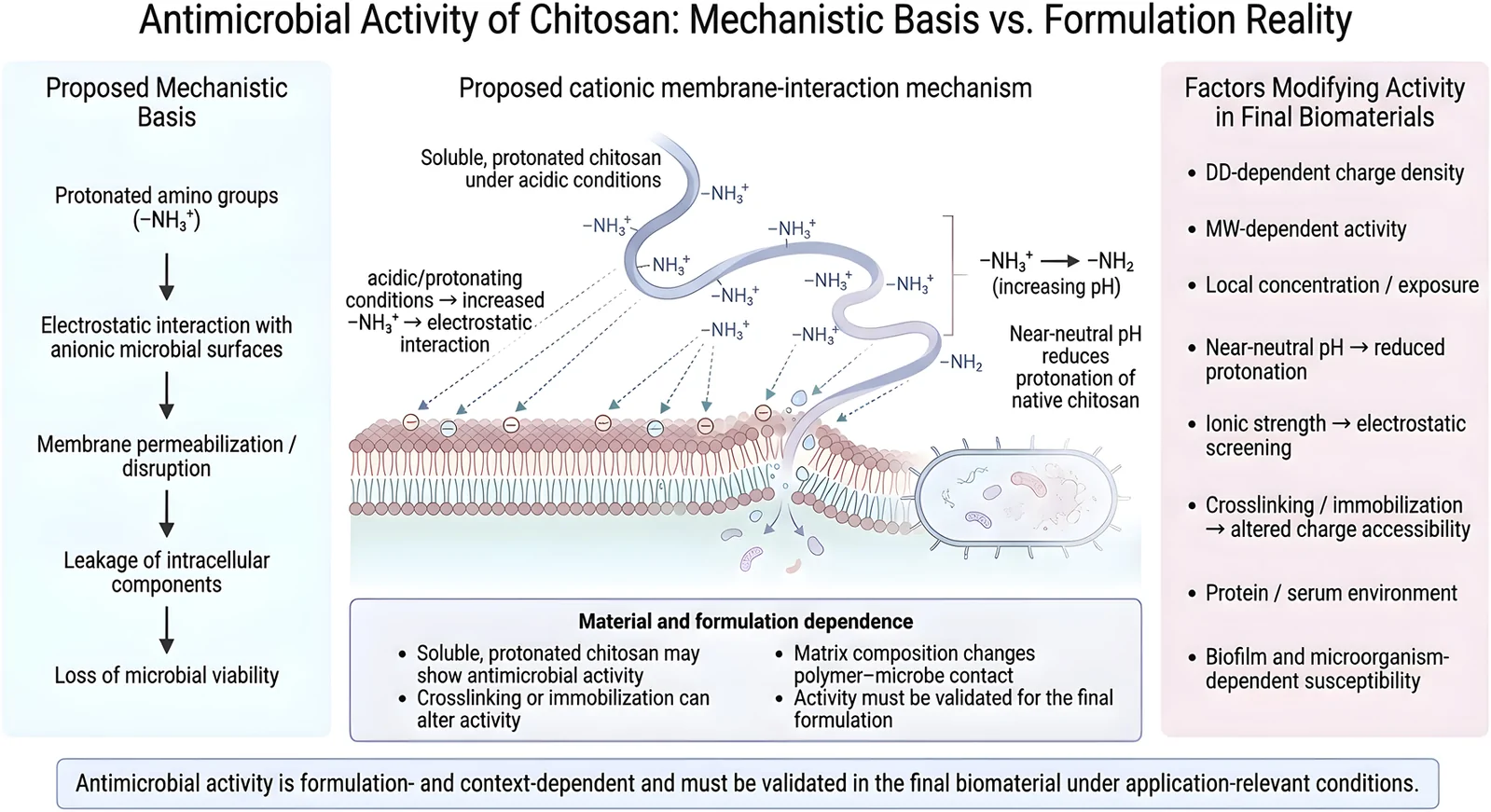
Antimicrobial activity of chitosan: from protonated polymer to formulated biomaterial. Soluble, protonated chitosan can interact electrostatically with microbial membranes and promote membrane disruption. In final materials such as hydrogels, xerogels, coatings, or composites, activity may be reduced by limited chain mobility, reduced amino-group accessibility, crosslinking, pH, ionic strength, additive shielding, or insufficient surface contact. This figure was prepared with the assistance of FigureLabs.ai.

### 2.8 Applications of chitosan in medical biomaterials and parameter-dependent performance

Chitosan is a versatile feedstock for diverse biomaterial formats, including tissue-engineering scaffolds, hydrogels, implant coatings, nanoparticle and microsphere drug-delivery systems, and bioactive dressings. In each format, the alignment of DD, MW, solubility, and purity with the intended functional requirement is decisive (Patrulea et al., 2015; Hamdi et al., 2025; Edo et al., 2024; Koirala et al., 2025; Mawazi et al., 2024; Qi et al., 2024).

For three-dimensional scaffolds used in bone, cartilage, or skin applications, raw-material parameters strongly influence performance. Such scaffolds are commonly produced by lyophilization, electrospinning, 3D printing, or casting of crosslinked sponges. MW mainly determines mechanical behavior. Longer chains generally increase strength, medium MW, approximately 100–300 kDa, can balance strength and processability, whereas very high MW increases viscosity and may impair uniform pore formation. DD modulates cell and protein interactions, with higher DD increasing charge density, adhesion, and protein adsorption, but also potentially increasing brittleness after hydration. Bone scaffolds commonly use chitosan with approximately 80%–90% DD, whereas DD values below 50% produce a more chitin-like and poorly wettable material (Sukpaita et al., 2021; Rodríguez-Vázquez et al., 2015; Cheung et al., 2015; Hamdi et al., 2025; Edo et al., 2024; Kumar et al., 2025; Ding et al., 2025; Gerova-Vatsova et al., 2025; Dinculescu et al., 2024).

DD and acetylation pattern also tune lysozyme-mediated degradation. In general, increasing DD reduces the abundance of acetylated sequences available for lysozyme-mediated cleavage, while higher MW can further reduce the rate of enzymatic degradation. However, acetylation pattern, crystallinity, crosslinking and scaffold architecture can substantially modify these trends. Consequently, long-term residence cannot be assigned to a specific DD range alone and should be determined for the final formulation under application-relevant conditions (Kumirska et al., 2010; Roman et al., 2020). However, biological responses remain parameter- and context-specific. Higher MW favored proliferation in one model, whereas lower MW improved bacterial penetration in another, indicating that there is no universal “best” chitosan (Hamdi et al., 2025; Koirala et al., 2025; Dhlamini et al., 2024; Ke et al., 2021; Teixeira-Santos et al., 2021).

To improve mechanical properties, chitosan scaffolds are often crosslinked, for example, with glutaraldehyde or genipin, or blended into composites such as chitosan-collagen and chitosan-alginate systems. Crosslinking depends on the availability of amino groups, and therefore on DD, while MW influences network density and processability. Rigorous reporting and standardization of these parameters are essential for reproducibility (Patrulea et al., 2015; Egorov et al., 2025; Hamdi et al., 2025; Koirala et al., 2025; Mawazi et al., 2024; Kumar et al., 2025).

Taken together, DD and MW should be considered as application-specific design variables rather than universal indicators of nterpre performance. Table 5 therefore provides a cross-application comparison of representative DD and MW ranges, degradation or residence nterpre, and the principal design trade-offs relevant to regenerative biomaterials. The values should be nterpreter as representative design windows rather than universal specifications, because degradation and functional performance are additionally governed by acetylation pattern, crystallinity, crosslinking, hydration, formulation composition, and final material architecture (Patrulea et al., 2015; Kumirska et al., 2010; Roman et al., 2020).

**TABLE 5:** Design-oriented comparison of representative degree of deacetylation (DD), molecular weight (MW), degradation behavior, and applications of chitosan-based regenerative biomaterials.

Biomaterial format/application	Representative DD	Representative MW	Representative degradation/residence behavior	Principal design implications	Key references
Wound dressings and hydroactive hydrogels	Typically ∼80–95% when high cationic charge, mucoadhesion or antimicrobial contribution is desired	Commonly ∼100–300 kDa for hydrogel-based systems	Short-term local residence; typically days for hydroactive formulations. Moderately crosslinked wound hydrogels are commonly designed to remain stable for several days. Actual degradation depends strongly on crosslinking and enzymatic exposure	Higher DD increases available amino groups and charge density; increasing MW and crosslink density improves cohesion but may reduce swelling and molecular diffusion	(Patrulea et al., 2015; Che et al., 2024; Guo et al., 2024; Koirala et al., 2025; Banach-Kopeć et al., 2022; Roman et al., 2020)
Injectable hydrogels and local drug/growth-factor depots	Generally moderate-to-high DD (∼80–90%) where efficient ionic or chemical network formation is required	Typically ∼100–300 kDa	Short-to-intermediate residence; formulation-dependent. Higher MW, higher DD and greater network density can reduce enzymatic erosion and slow molecular diffusion, whereas loosely crosslinked systems degrade or release cargo more rapidly	Medium MW balances injectability and network cohesion. High DD can strengthen TPP-mediated or other ionic crosslinking but may increase stiffness, brittleness or premature gelation	(Muzzarelli, 2009; Uddin et al., 2024; Che et al., 2024; Edo et al., 2024; Koirala et al., 2025; Wang and Roman, 2023; Roman et al., 2020; Salamat et al., 2025)
Porous 3D scaffolds for bone, cartilage and skin	Frequently ∼80–90% in scaffold formulations; DD should be selected together with acetylation pattern rather than alone	Frequently ∼100–300 kDa; higher MW may be used where greater structural stability is required	Intermediate-to-long residence, commonly designed for weeks to months depending on tissue-repair window. Enzymatic persistence generally increases with reduced lysozyme susceptibility, dense crosslinking and restricted enzyme access	Higher MW improves chain entanglement and mechanical integrity but excessive MW impairs mixing and uniform pore formation. DD influences protein interactions, crosslinking and enzymatic susceptibility	(Sukpaita et al., 2021; Rodríguez-Vázquez et al., 2015; Patrulea et al., 2015; Kumirska et al., 2010; Roman et al., 2020; Kumar et al., 2025; Elizalde-Cárdenas et al., 2024; Tupe et al., 2024; Piszko et al., 2024)
Implant coatings and layer-by-layer films	Usually moderate-to-high DD; ∼80–95% where strong surface charge and interaction with anionic components are required	Approximately 50–150 kDa for many LbL systems; broader coating formulations ∼50–300 kDa	Surface-retention dominated and highly formulation-specific. One reported partially depolymerized chitosan coating system resorbed over approximately 2 weeks; coating persistence should otherwise be verified for the final device	Higher DD strengthens electrostatic interactions and may enhance antibacterial surface activity, whereas very high DD or MW can reduce solubility and complicate uniform film formation	(Koirala et al., 2025; von Seggern et al., 2025; Dhlamini et al., 2024; Ke et al., 2021; Teixeira-Santos et al., 2021; Savdenbekova et al., 2024; Ghimire et al., 2025; Villegas et al., 2022)
Bioinks and 3D-bioprinted constructs	Commonly moderate-to-high DD (∼80–90%), depending on the secondary polymer and crosslinking mechanism	Commonly ∼100–300 kDa; >∼500 kDa may impair extrusion	Tissue-maturation-matched degradation; no universal rate. Residence is controlled predominantly by crosslinking chemistry, polymer partners and printed architecture rather than MW or DD alone	Higher MW improves viscosity and filament fidelity but increases extrusion resistance and cell shear stress. Higher DD strengthens interactions with polyanions and can improve shape fidelity, but excessive reactivity may cause premature gelation	(Banach-Kopeć et al., 2022; Salamat et al., 2025; Sauce-Guevara et al., 2025; Le Ba Thai et al., 2024; Bi et al., 2020; Mathur et al., 2025; Reberte et al., 2025)
Nanoparticles and nanoscale drug-delivery systems	Commonly moderate-to-high DD, selected to provide sufficient cationic charge for ionic complexation and cargo interaction	Low-to-medium MW; formulation-specific. Oligomeric/near-oligomeric fractions <∼10 kDa provide greater solubility and reactivity, whereas higher MW produces denser structures	Generally faster for low-MW/less densely crosslinked systems and slower with increasing chain length and network density; quantitative rates are formulation- and assay-dependent	Lower MW facilitates dissolution, diffusion and processing; increasing MW or crosslinking can increase retention and prolong release but may decrease cargo diffusion	(Pereira Parchen et al., 2025; Koirala et al., 2025; Pudžiuvelytė et al., 2022; Al-Jbour et al., 2019; Roman et al., 2020; Salamat et al., 2025)

## 3 Chitosan-based hydrogels and injectable materials

Chitosan hydrogels are three-dimensional, water-swollen networks used as hydroactive wound dressings, drug-delivery systems, and injectable implants for minimally invasive repair (Che et al., 2024; Salamat et al., 2025). They can form through physical interactions, including temperature- or pH-driven sol-gel transitions, such as neutralization of acidic chitosan solutions with basic phosphate, or through chemical crosslinking. Multivalent anions, such as tripolyphosphate (TPP), can also induce ionic gelation, a mechanism widely used both for hydrogel formation and for the preparation of chitosan nanoparticles (Pereira Parchen et al., 2025; Egorov et al., 2025; Salamat et al., 2025).

Polymer parameters govern hydrogel rheology, gelation kinetics, and degradation. High-MW chitosan forms cohesive gels, but can hinder mixing and injectability. For this reason, medium-MW chitosan, approximately 100–300 kDa, is often preferred for injectable systems. Higher DD increases the number of protonated amino groups and can increase crosslink density, leading to faster and stiffer TPP-based gelation, although such gels may also be more brittle. Lower-DD analogues usually form softer and more elastic, but mechanically weaker, networks (Hamdi et al., 2025; Edo et al., 2024; Koirala et al., 2025; Wang and Roman, 2023; Salamat et al., 2025).

In wound care, chitosan hydrogels can maintain a moist environment in chronic or hard-to-heal wounds, including burns and diabetic ulcers. They can also absorb exudate and provide antibacterial or pro-healing functionality, depending on formulation. These applications typically require moderately crosslinked, elastic gels that adhere to the wound, remain stable for several days, and can be removed without additional tissue damage (Che et al., 2024; Guo et al., 2024; Banach-Kopeć et al., 2022).

As drug carriers, chitosan hydrogels can control the release of antibiotics, cytostatics, and growth factors. Stronger crosslinking and higher DD or MW generally slow diffusion, whereas lower-DD and more hydrophobic chitosans may bind hydrophobic drugs and prolong release. This tunability enables profiles such as gradual growth-factor release in angiogenic wound-healing gels (Che et al., 2024; Koirala et al., 2025; Salamat et al., 2025).

Animal models support the regenerative potential of selected chitosan hydrogels compared with controls. For example, carboxymethyl chitosan dressings have been reported to reduce scarring and accelerate burn re-epithelialization. Overall, native chitosan, its derivatives, including N,O-carboxymethyl and trimethyl chitosan, and tuned crosslink densities provide a versatile hydrogel toolkit for wound care, drug delivery, and minimally invasive regenerative applications (Che et al., 2024; Guo et al., 2024; Egorov et al., 2025; Verma and Reddy, 2024; Salamat et al., 2025).

### 3.1 Chitosan coatings and surfaces

Chitosan coatings can act as bioactive and antibacterial layers on implants, as well as nanoscale layer-by-layer (LbL) assemblies on stents and culture scaffolds (Teixeira-Santos et al., 2021; Savdenbekova et al., 2024). As an adhesive polycation, chitosan can be deposited on metals, glass, and polymers to form thin films. On titanium, such coatings have been reported to improve osteoblast adhesion and provide antibacterial activity (Ghimire et al., 2025; Villegas et al., 2022).

Material parameters determine coating stability and function. MW influences film thickness and continuity, with higher-MW chitosan generally producing more continuous but thicker films. In LbL systems, chitosan with MW values of approximately 50–150 kDa is often used to obtain nanometric layers. DD determines surface charge density, with higher DD providing more -NH_3_^+^ groups that can strengthen electrostatic interactions with microbial membranes. However, coatings are generally more bactericidal against Gram-positive than Gram-negative species, and MW-dependent activity trends remain strain- and model-dependent. MW must therefore be sufficient to resist erosion, while DD should support antimicrobial performance without excessive biological activation (Egorov et al., 2025; Dhlamini et al., 2024; Ke et al., 2021; Teixeira-Santos et al., 2021; Savdenbekova et al., 2024).

The required balance also depends on the implant setting. On vascular stents, moderate surface charge may support controlled platelet interactions and antithrombotic sealing, whereas excessive DD can promote aggregation. On orthopedic hardware, chitosan coatings can carry growth factors such as bone morphogenetic protein (BMP-2) or TGF-β. In this context, higher DD may favor binding of anionic proteins, while sufficient MW helps resist premature dissolution (Egorov et al., 2025; Teixeira-Santos et al., 2021; Savdenbekova et al., 2024).

*In vivo* studies illustrate the application-specific nature of these effects. Chitosan-coated titanium screws showed faster osseointegration, reduced biofilm formation, and maintained biomechanics compared with bare titanium, with nano-hydroxyapatite further improving outcomes. In another context, partially depolymerized chitosan suture coatings released fragments and resorbed over approximately 2 weeks, matching the re-epithelialization window (Koirala et al., 2025; Teixeira-Santos et al., 2021; Ghimire et al., 2025).

### 3.2 Practical design conclusions

The practical implications of these parameters can be consolidated into a translational decision framework. In this framework, source selection, DD and MW specification, purity and endotoxin control, solvent system, crosslinking strategy, and sterilization are matched to the intended clinical function rather than chosen independently.

In general, higher DD values, approximately 80%–95%, favor solubility, cationic charge, mucoadhesion, and antimicrobial activity, making them relevant for wound dressings, coatings, and gene-delivery systems. For applications requiring prolonged structural persistence, higher MW together with controlled crosslinking and sufficiently high DD may reduce enzymatic erosion; however, residence time should be experimentally established for the final formulation because acetylation pattern, crystallinity and material architecture can substantially modify DD- and MW-dependent degradation (Kumirska et al., 2010; Roman et al., 2020). Regardless of application, purity remains a non-negotiable baseline, particularly for implantable materials, where endotoxin levels should be below 0.1 EU/mg and verified using LAL or rFC assays (Patrulea et al., 2015; Egorov et al., 2025; Koirala et al., 2025; Reay et al., 2023). A full minimum reporting set is provided in the Conclusions. This framework is summarized in Figure 6.

**FIGURE 6:**
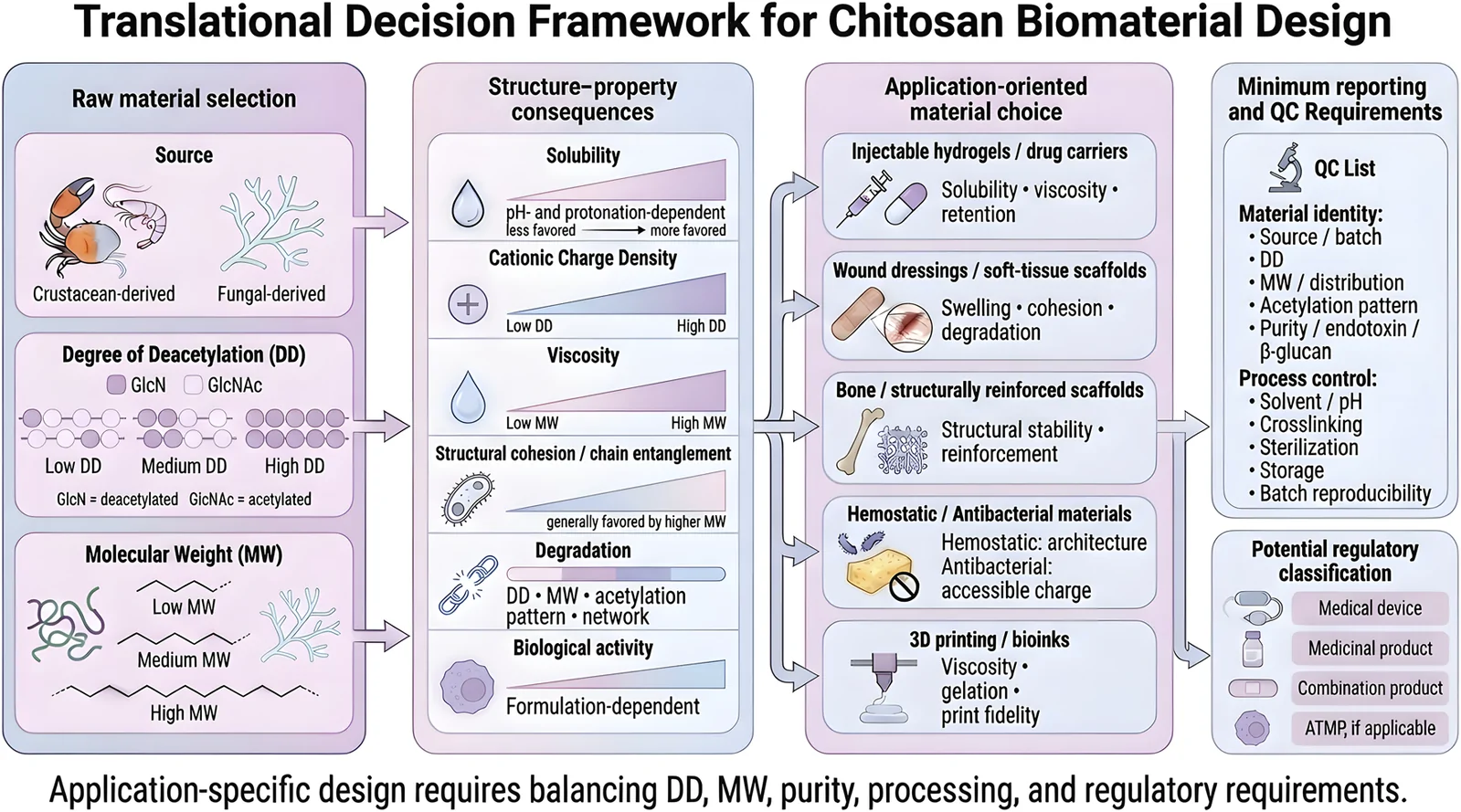
Translational decision framework for chitosan-based biomaterial design. Selection of source, DD, MW, purity, solvent system, crosslinking strategy, and sterilization method should be driven by intended clinical function. This framework links raw-material specifications and quality-control requirements with application-specific needs, including wound dressings, injectable hydrogels, hemostatic materials, coatings, scaffolds, and bioinks. This figure was prepared with the assistance of FigureLabs.ai.

## 4 Functionalization and composites of chitosan in tissue engineering

Chitosan’s processability and tunable chemistry make it widely used in drug delivery, tissue-engineering scaffolds, and growth-factor delivery, with MW and DD strongly shaping how it is functionalized and how it interacts with biological systems (Elizalde-Cárdenas et al., 2024; Egorov et al., 2025). This section critically reviews targeted functionalization and composite design for regenerative medicine.

### 4.1 Growth factors, drugs, and extracellular vesicles

Functionalization transforms chitosan from a structural matrix into a delivery and signaling platform; the central challenge is to preserve cargo bioactivity while matching release, retention, and presentation to the biological sequence of repair.

#### 4.1.1 Growth factors

##### 4.1.1.1 Delivery strategies and mechanistic tradeoffs

Growth-factor functionalization of chitosan biomaterials should be evaluated as a problem of protein presentation, not simply protein loading. Most regenerative growth factors are dose-sensitive, structurally fragile, and context-dependent. BMP-2, VEGF, fibroblast growth factor (FGF-2), platelet-derived growth factor (PDGF), nerve growth factor (NGF), and related factors can produce desirable effects within a narrow spatiotemporal window but may cause ectopic tissue formation, aberrant vascularization, inflammation, fibrosis, or loss of efficacy if released in an uncontrolled burst. Chitosan’s cationic and hydrogel-forming properties can be useful, but only when the formulation protects the protein, controls its release, preserves bioactivity, and places the dose within a clinically plausible range (Sa et al., 2012; Yu et al., 2018; Gan et al., 2018; Obara et al., 2003).

The relevant design question is therefore not whether a chitosan scaffold can be loaded with a growth factor. It is whether the strategy controls (i) loading efficiency, (ii) burst release, (iii) protein denaturation during loading and sterilization, (iv) displacement by serum proteins, (v) diffusion through the scaffold, (vi) degradation-coupled liberation, and (vii) post-release bioactivity. Several chitosan-based growth-factor delivery studies have characterized cumulative release under simplified PBS conditions (Gan et al., 2018; Zhang et al., 2024), whereas direct assessment of the structural integrity or specific biological activity of the released protein has received considerably less attention. Such release data demonstrate formulation feasibility but, in the absence of post-release potency testing, do not by themselves establish a regenerative mechanism.

##### 4.1.1.2 Adsorption

Adsorption is experimentally simple and often increases apparent loading because proteins interact with chitosan through electrostatic and hydrogen-bonding interactions. However, it is also the least controlled approach. Growth factors adsorbed to a cationic surface can desorb rapidly, become partially denatured, or be displaced by serum proteins once exposed to wound exudate, plasma, synovial fluid, or interstitial fluid. Adsorption-only designs are most defensible when the intended clinical need is short-term local exposure and when burst release is acceptable. They are less defensible for BMP-2, VEGF, or NGF delivery where uncontrolled early release can reduce efficacy or increase adverse-effect risk (Sa et al., 2012).

##### 4.1.1.3 Ionic complexation and affinity-based retention

Ionic complexation exploits the cationic nature of chitosan and the charge properties of proteins or polyanions to form polyelectrolyte complexes under mild aqueous conditions; the limitation is sensitivity to pH, salt concentration, competing proteins, and local degradation, so complexes stable in low-ionic-strength buffer may release rapidly under physiological conditions. This approach becomes more rational when the binding interaction is designed around the growth factor’s natural biology: for heparin-binding factors, incorporating heparin, heparan-sulfate-like domains, or sulfated chitosan can mimic extracellular matrix retention, moving the formulation from nonspecific electrostatics toward biologically informed affinity retention (Li et al., 2017; Yu et al., 2018).

Heparin/chitosan systems are particularly relevant for NGF, VEGF, FGF-2, and other heparin-binding proteins, aligning release control with growth-factor biochemistry rather than nonspecific adsorption; a heparin/chitosan-NGF scaffold, for example, improved peripheral nerve regeneration in preclinical models (Li et al., 2017). Sulfated chitosan extends this logic by chemically converting chitosan itself into a heparin-mimetic material: 2-N,6-O-sulfated chitosan can manipulate VEGF-induced angiogenesis, a more mechanistically precise claim than “chitosan is pro-angiogenic,” since the active material is a sulfated derivative whose effect depends on sulfation position, charge density, MW, and VEGF interaction (Yu et al., 2018).

Affinity systems can reduce burst release and protect growth factors, but introduce safety and manufacturing questions: heparin source, anticoagulant activity, sulfation heterogeneity, batch consistency, and residual reagents must be controlled, and for sulfated chitosan, substitution pattern and degree of sulfation become critical quality attributes. These systems are translationally more plausible than adsorption-only platforms but require more sophisticated analytics and release-potency assays (Yu et al., 2018).

##### 4.1.1.4 Affinity-based retention

Heparin/chitosan systems are particularly relevant for NGF, VEGF, FGF-2, and other heparin-binding proteins. Heparin can bind and stabilize growth factors, while chitosan contributes scaffold formation, processability, and tissue adhesion. A heparin/chitosan scaffold loaded with NGF was designed to exploit electrostatic retention and improve peripheral nerve regeneration in preclinical models (Li et al., 2017). The conceptual strength of such a system is that it aligns release control with growth-factor biochemistry rather than relying on nonspecific adsorption.

Sulfated chitosan extends this logic by chemically modifying chitosan itself into a heparin-mimetic material. A study of 2-N,6-O-sulfated chitosan showed that sulfation pattern can manipulate VEGF-induced angiogenesis, suggesting that modified chitosan can influence growth-factor signaling, not merely release kinetics (Yu et al., 2018). This is a more mechanistically precise claim than “chitosan is pro-angiogenic.” The active material is a sulfated derivative with growth-factor-binding features, and its biological effect should be interpreted as a function of sulfation position, charge density, MW, and VEGF interaction.

Affinity systems can reduce burst release and protect growth factors, but they also introduce safety and manufacturing questions. Heparin source, anticoagulant activity, sulfation heterogeneity, batch consistency, and residual reagents must be controlled. For sulfated chitosan, substitution pattern and degree of sulfation become critical quality attributes. Translationally, these systems are more plausible than adsorption-only platforms but require more sophisticated analytics and release-potency assays (Yu et al., 2018).

##### 4.1.1.5 Encapsulation and nanoparticle-in-scaffold systems

Encapsulation places the growth factor inside particles, microspheres, or hydrogel domains that are then integrated into a scaffold. This can reduce burst release, protect the protein during early exposure, and enable staged delivery. Chitosan/biphasic calcium phosphate scaffolds functionalized with BMP-2-encapsulated nanoparticles and arginine-glycine-aspartate (RGD) peptides illustrate a multilayer strategy: the mineral phase contributes osteoconductivity, RGD supports adhesion, and nanoparticles regulate BMP-2 presentation (Gan et al., 2018). The outcome should therefore be interpreted as a composite effect, not as a chitosan-only osteogenic response.

Nanoparticle-in-scaffold systems can also separate multiple biological phases. For infected bone defects, an antibacterial phase can precede an osteogenic phase; for wound healing, early hemostasis or antimicrobial action can be followed by angiogenic and matrix-remodeling cues. However, staged release is difficult to prove unless the study measures time-resolved release of each cargo under relevant conditions and demonstrates that each released cargo remains active.

##### 4.1.1.6 Covalent immobilization

Covalent immobilization can improve spatial retention and reduce burst release and is particularly useful when sustained matrix-bound signaling rather than diffusional delivery is desired (Kiryk et al., 2025; Budiraharjo et al., 2013; Karakeçili et al., 2008; Ham et al., 2017). In chitosan-based systems, covalent growth-factor presentation has been demonstrated using several coupling strategies. BMP-2 and FGF-2 were immobilized on chitosan films through EDC/NHS-mediated coupling, with the immobilized factors retaining biological activity and promoting osteoblast- or fibroblast-associated responses compared with adsorbed growth factors (Budiraharjo et al., 2013). EGF has also been photochemically immobilized on chitosan membranes using a heterobifunctional crosslinker, where the immobilized factor stimulated fibroblast proliferation (Karakeçili et al., 2008). Covalent growth-factor tethering has additionally been applied in methacrylamide-chitosan hydrogel systems to direct neural stem-cell differentiation and self-organization (Ham et al., 2017).

However, covalent attachment is not intrinsically advantageous. Coupling may alter protein conformation or modify residues involved in receptor recognition, and successful immobilization should therefore not be equated with retained biological activity (Kiryk et al., 2025; Budiraharjo et al., 2013; Karakeçili et al., 2008; Ham et al., 2017). Consequently, covalently immobilized growth factors should be evaluated using receptor-binding or cell-based functional assays in the final material configuration, particularly after immobilization, sterilization, and storage.

##### 4.1.1.7 Degradation-coupled release

Degradation-coupled release is attractive for regenerative medicine because it ties cargo liberation to scaffold residence and tissue remodeling. A photocrosslinkable chitosan hydrogel containing FGF-2 demonstrated biologically active release upon degradation and improved wound healing in diabetic mice, providing an early example of this logic (Obara et al., 2003). Although older, this study remains conceptually important because it did not treat release kinetics as a purely physical diffusion problem; it linked release to biodegradation and tested biological activity.

Across all delivery strategies described above, the methodological baseline is the same: release should be measured in protein- and enzyme-containing media rather than PBS alone, early time-point resolution matters as much as cumulative release, and post-release bioactivity must be confirmed with a growth-factor-specific assay rather than inferred from downstream gene expression. These controls, together with the translational trade-offs of each strategy, are summarized in Table 4.

##### 4.1.1.8 Evidence for regenerative outcomes in wound healing of distinct tissues/organs

Bone repair is the most common setting for BMP-2-functionalized chitosan composites. Recent systems increasingly combine antibacterial and osteogenic delivery, reflecting the clinical reality that infection and inflammation can block bone regeneration. A staged ciprofloxacin/BMP-2 nanoparticle system in a chitosan-containing scaffold illustrates this infection-first, regeneration-second design logic (Zhang et al., 2024). Similar sequential vancomycin/BMP-2 platforms for chronic osteomyelitis extend the concept to infected bone defects, although these remain preclinical and require careful infection-model validation (Zhan et al., 2024).

Wound healing systems frequently use FGF-2, PDGF, VEGF, bFGF, or combinations thereof. A PDGF-AA-loaded photo-crosslinked chitosan-based hydrogel reported improved handling, hemostatic properties, and wound-healing-associated cellular behaviors (Obara et al., 2005). Dual growth-factor systems such as VEGF plus bFGF attempt to reproduce the temporal complexity of wound healing, but mechanistic claims require proof that each factor remains bioactive after loading and release (Chevrier et al., 2017).

Peripheral nerve repair systems often use NGF retention through heparin/chitosan scaffolds. The biological rationale is stronger than adsorption-only approaches because NGF is a heparin-binding neurotrophic factor and Schwann-cell behavior depends on local trophic support (Li et al., 2017). Still, preclinical nerve-repair outcomes should be compared with clinically relevant nerve conduits and autograft benchmarks. A chitosan-NGF scaffold that improves a rat nerve gap model is not automatically clinically competitive.

Cartilage repair research has seen a rise in the utilization of chitosan hydrogels in combination with chondrogenic small molecules, growth factors, EVs or cells. Within these systems, chitosan is often used for its ability to provide a local retention and mechanical environment rather than serving as the main chondrogenic signal. It is important for claims to distinguish between the contribution of the scaffold itself and the cargos it may contain. Furthermore, assessments should include mechanical testing, analysis of matrix quality, and consideration of the inflammatory context, rather than solely relying on the expression of chondrogenic ECM components.

#### 4.1.2 Drugs

##### 4.1.2.1 Strategy-by-format analysis

Drug functionalization of chitosan biomaterials includes small molecules, antibiotics, anti-inflammatory agents, osteogenic drugs, analgesics, and antiseptic compounds. The design logic differs from growth-factor delivery because many drugs are more chemically stable, less structurally fragile, and easier to quantify. Nevertheless, the same translational questions apply: release in relevant fluids, dose realism, sterilization stability, storage, local toxicity, resistance risk for antibiotics, and compatibility with tissue repair.

Hydrogels are suited for injectable depots and irregular defects because they can fill space, retain water, and release drug as the network swells or degrades. Films and membranes are suited for topical wounds and periodontal applications. Porous scaffolds and 3D-printed composites are more relevant for bone defects where mechanical support and local delivery are needed. Nanoparticles or microparticles within scaffolds can add a rate-limiting barrier and reduce burst release (Gao et al., 2023; Boroojen et al., 2019).

##### 4.1.2.2 Hydrogels and injectable depots

Chitosan hydrogels can act as local depots through ionic interactions, mesh-size restriction, swelling control, and degradation. Thermosensitive systems, genipin-crosslinked gels, beta-glycerophosphate formulations, carboxymethyl chitosan gels, and methacrylated chitosan networks all have different release mechanisms. Therefore, “chitosan hydrogel release” should not be treated as one technology.

The critical limitation is that many hydrogel studies use release media that do not represent clinical conditions. PBS release without proteins, enzymes, pH variation, inflammatory mediators, or mechanical compression may overestimate stability or underestimate burst release. For wound, bone infection, and cartilage applications, release should be tested in media that approximate exudate, infected tissue fluid, or synovial-like environments, followed by antimicrobial, anti-inflammatory, or osteogenic potency testing of the released fraction.

Infected bone defects illustrate why drug delivery and regeneration cannot be separated. Local antibiotics may reduce bacterial burden, but excessive local concentrations can impair osteoblasts, MSCs, endothelial cells, or immune resolution. Conversely, growth factors delivered before infection control may fail or worsen inflammation. Chitosan-based staged systems that release an antibiotic first and an osteogenic cue later are therefore biologically plausible (Zhang et al., 2024; Zhan et al., 2024).

A 3D-printed polylactic acid (PLA)/nano-hydroxyapatite scaffold incorporating a chitosan-vancomycin hydrogel reported sustained vancomycin release over weeks, inhibition of *Staphylococcus aureus*, and osteoblast compatibility (Gao et al., 2023). The design is clinically relevant because local antibiotic delivery can achieve concentrations difficult to maintain systemically. However, translation requires *in vivo* infection models, biofilm relevance, resistance monitoring, local cytotoxicity thresholds, and comparison with established local-delivery standards.

Anti-inflammatory drugs, corticosteroids, non-steroidal agents, and osteogenic small molecules can be incorporated into chitosan nanoparticles, hydrogels, or fibers. Dexamethasone sodium phosphate-loaded chitosan nanoparticles embedded in electrospun scaffolds provide an example of nanoparticle-mediated rate control within a composite scaffold (Boroojen et al., 2019).

#### 4.1.3 Extracellular vesicles (EVs)

##### 4.1.3.1 Correct EV terminology

The EV field has moved away from the casual use of biogenesis-based terms such as “exosomes” unless their endosomal origin has been directly demonstrated. The 2018 and 2023 *Minimal Information for Studies of Extracellular Vesicles* guidelines recommend using “extracellular vesicles” as a generic term, or more precise operational descriptors such as “small EVs,” “large EVs,” “CD63-positive EVs,” or “cell-derived EVs,” depending on the separation method, size range, molecular markers, and cellular source (Théry et al., 2018; Welsh et al., 2024). This distinction is particularly important for chitosan-based biomaterials, because many studies incorporate EV-enriched or extracellular particle preparations into hydrogels or scaffolds and then attribute the observed biological effects to EVs without sufficient evidence of vesicular identity, purity, or functional specificity.

Chitosan platforms may amplify this interpretive problem. Due to its cationic and adsorptive nature, chitosan can retain not only EVs, but also protein aggregates, lipoproteins, nucleic acid-protein complexes, serum-derived particles, adsorbed growth factors, and other non-vesicular extracellular particles. Therefore, biological effects observed after release from chitosan-based matrices may result from EVs themselves, co-isolated soluble factors, serum contaminants, adsorbed bioactive molecules, or matrix-dependent release kinetics. For this reason, EV-loaded chitosan systems should be described operationally and reported with sufficient methodological detail, including the source cell type, culture conditions, serum or serum-free protocol, EV separation method, size fraction, marker characterization, purity metrics such as particle-to-protein ratio, potency assay, loading strategy, release method, and post-release EV integrity (Théry et al., 2018; Welsh et al., 2024; Lener et al., 2015).

Fetal bovine serum (FBS) contamination is a major limitation in EV studies. Even EV-depleted serum may contain residual bovine EVs, extracellular RNA, proteins, lipoproteins, and other contaminants that can confound the interpretation of human or animal EV preparations (Wu et al., 2024; Urzì et al., 2022; Mannerström et al., 2019). Donor and source variability is also scientifically and ethically relevant. MSC-derived EVs can differ according to tissue source, donor age, disease state, passage number, culture stress, and manufacturing conditions. These variables should therefore be reported and, where possible, controlled or acknowledged as limitations.

##### 4.1.3.2 EV loading strategies in chitosan biomaterials

Physical entrapment, which disperses EVs within a gel precursor before gelation, is the most common EV loading strategy because it avoids harsh conjugation chemistry and suits injectable hydrogels and dressings. However, the EV preparation is exposed to the gelation environment—shear, pH, crosslinking chemistry, photoinitiators, elevated temperature, or osmotic shifts can alter EV membrane integrity or surface proteins—so post-loading integrity must be measured rather than assumed from pre-loading size distribution alone, and released EVs should be tested for particle number, protein markers, non-vesicular contaminants, and potency (Welsh et al., 2024). A chitosan hydrogel dressing loaded with adipose MSC-derived vesicles, for example, reported slow release associated with chitosan degradation and improved wound repair in a rat model (Wu et al., 2024), though interpretation depends on whether EV identity was operationally confirmed rather than assumed.

Electrostatic adsorption can improve EV retention on chitosan fibers or particles, but may also mask surface molecules, promote aggregation, co-adsorb non-vesicular proteins, or alter biodistribution and receptor presentation: positively charged chitosan can bind negatively charged EV membranes and co-isolated proteins into aggregates that release differently from free EVs, so release curves expressed only as particle counts can be misleading unless protein release, vesicle integrity, and potency are separately assessed.

Hydrogel retention and scaffold integration are translationally attractive because local EV delivery may avoid rapid clearance after injection, but the relevant potency is that of the released fraction at each time point rather than of the starting suspension—a scaffold that retains EVs too strongly may show excellent loading but poor bioavailability. This complexity increases when EVs are combined with cells: an nHA/chitosan/poly (lactic-co-glycolic acid) (PLGA) scaffold delivering adipose stem cell-derived EVs and bone marrow stem cells for bone healing is a multi-component product in which osteogenesis could arise from mineral phase, cells, EVs, or their interactions (Wan et al., 2024), requiring factorial controls (scaffold alone, plus cells, plus EVs, plus both) to attribute outcomes correctly.

Hybrid systems can assign distinct functions to distinct components: an immunomodulatory EV-loaded chitosan methacryloyl hydrogel combined with pro-chondrogenic microspheres frames EVs as immune microenvironment modulators and kartogenin-containing microspheres as chondrogenic drivers (Liang et al., 2025), a division of labor more mechanistically defensible than attributing all repair to EVs—although using vesicles from LPS-activated macrophages raises additional safety questions around endotoxin carryover and residual activation signals.

##### 4.1.3.3 Modulation of EV retention in biomaterials

Affinity-based retention via heparin/chitosan systems or sulfated chitosan is more mechanistically justified than simple adsorption, particularly for heparin-binding growth factors. Sulfated chitosan can modulate VEGF-induced angiogenesis, while heparin/chitosan scaffolds can retain NGF in nerve-repair applications (Li et al., 2017; Yu et al., 2018). Adsorption-only loading is less convincing translationally unless burst release, protein displacement, and post-release bioactivity are controlled (Sa et al., 2012). Similarly, composite systems combining antibacterial and osteogenic functions may be advantageous in infected bone defects (Zhang et al., 2024; Gao et al., 2023; Zhan et al., 2024), but release studies performed only in PBS may poorly reflect protein-rich, enzymatic, inflammatory, or mechanically loaded *in vivo* environments. Sterilization and storage can further alter chitosan network properties and release behavior, supporting post-sterilization evaluation of release kinetics and bioactivity (Szymańska and Winnicka, 2015).

For EV-loaded chitosan systems, retention and release should be interpreted in accordance with the 2018 and 2023 *Minimal Information for Studies of Extracellular Vesicles* guidelines (Théry et al., 2018; Welsh et al., 2024). Inadequate demonstration of vesicle identity, purity, or functional specificity may confound biological outcomes through non-EV fractions, serum-derived contaminants, co-isolated proteins, or chitosan-mediated retention effects (Théry et al., 2018; Welsh et al., 2024; Urzì et al., 2022; Mannerström et al., 2019).

The functionalization strategies discussed throughout this section, together with their key advantages, limitations, and translational controls, are summarized in Table 3.

### 4.2 Peptide functionalization of chitosan-based biomaterials

#### 4.2.1 Why functionalize biomaterials with peptides?

Chitosan can be cytocompatible and antimicrobial in suitable formulations, but it lacks the adhesive signaling motifs of the extracellular matrix (ECM), which can limit cell adhesion and differentiation (Mochizuki et al., 2007). For this reason, chitosan surfaces or bulk structures are often modified with short ECM-derived peptides, particularly the integrin-binding Arg-Gly-Asp (RGD) sequence, to provide receptor-mediated cues that guide adhesion, proliferation, migration, and differentiation (Brun et al., 2021; Mochizuki et al., 2007; Forero et al., 2023).

Peptide functionalization can also be used to deliver pro-angiogenic or antimicrobial peptides. When incorporated into chitosan nanoparticles or matrices, these peptides can be released gradually, prolonging their local bioactivity (Pereira Parchen et al., 2025; Hozumi and Nomizu, 2018; Kiryk et al., 2025) (Table 4). Thus, peptide incorporation serves two complementary purposes: improving cell affinity through adhesive motifs and providing sustained biological signaling through therapeutic peptides. For example, RGD-modified chitosan improves cell attachment and proliferation, whereas controlled release of therapeutic peptides can support prolonged regenerative signaling (Brun et al., 2021; Elizalde-Cárdenas et al., 2024; Mochizuki et al., 2007; Forero et al., 2023; Hozumi and Nomizu, 2018; Kiryk et al., 2025).

#### 4.2.2 Influence of molecular weight and degree of deacetylation on peptide conjugation

Chitosan is a linear copolymer of glucosamine and N-acetylglucosamine whose functionalization depends mainly on MW and DD. Low-MW chitosan (here referring to oligomeric and near-oligomeric fractions, approximately below 10 kDa, at the lower boundary of the commercial MW range discussed above) dissolves and reacts more readily and can support peptide encapsulation, but it tends to form smaller and less dense particles. In contrast, high-MW chitosan hydrogels form denser networks that slow peptide diffusion and prolong release (Hamdi et al., 2025; Salamat et al., 2025).

High DD, approximately 90%–95%, increases the density of protonatable amino groups and provides a greater number of primary amines available for peptide coupling. In contrast, lower DD reduces the number of available amino groups and may therefore limit the extent of covalent peptide attachment (Egorov et al., 2025; von Seggern et al., 2025; Dinculescu et al., 2024; Elizalde-Cárdenas et al., 2024).

These parameters determine the coupling mechanism. In physical encapsulation, peptides are retained through electrostatic or physical entrapment. Low MW and moderate DD may favor higher porosity and faster release, whereas dense high-MW networks are more suitable for prolonged release (Pereira Parchen et al., 2025; Salamat et al., 2025). In covalent immobilization, peptides are typically linked to free amine groups on chitosan using 1-ethyl-3-(3-dimethylaminopropyl)carbodiimide/N-hydroxysuccinimide (EDC/NHS) chemistry. Higher DD provides more reactive amines, while high MW supports a stable network that can prolong signal presentation. Cells generally distinguish adsorbed from covalently bound peptides and often respond more strongly to immobilized cues. However, DD must be optimized, because partial acetylation may leave too few available amines, while low-pH protonation can promote swelling or layer detachment (Egorov et al., 2025; Brun et al., 2021; Forero et al., 2023).

In practice, the choice depends on peptide function, material format, and peptide stability. Slow release is usually preferable for soluble signaling peptides, whereas surface presentation is more appropriate for adhesive peptides such as RGD or serine-isoleucine-lysine-valine-alanine-valine (SIKVAV). Immobilization is typically favored in dense xerogels and coatings, while both encapsulation and immobilization can be used in soft hydrogels and bioinks. Chitosan matrices may also protect peptides from enzymatic degradation more effectively than solution-based delivery (Hamdi et al., 2025; Koirala et al., 2025; Savdenbekova et al., 2024; Ghimire et al., 2025; Hozumi and Nomizu, 2018; Kiryk et al., 2025).

#### 4.2.3 Free versus covalently bound peptides in different biomaterial forms

The choice between free and bound peptide presentation should match the mode of action, exposure time, and material format. In hydrogels, free peptides can act as depot-based signals, because peptide-polymer interactions slow diffusion and help sustain local signaling concentrations. In contrast, adhesive motifs such as RGD are often best covalently immobilized, for example, using EDC/NHS chemistry, to prevent leaching and control ligand density and orientation. Covalently anchored sequences such as SIKVAV have been shown to enhance fibroblast proliferation and wound healing (Pereira Parchen et al., 2025; Brun et al., 2021; Salamat et al., 2025; Elizalde-Cárdenas et al., 2024; Forero et al., 2023).

In xerogels, rehydration can readily wash out free peptides, making covalent conjugation the more robust strategy. High DD provides amines for coupling, while higher MW helps stabilize the matrix during wet-dry cycles (Hamdi et al., 2025; Brun et al., 2021). In implant coatings, surface presentation is suitable for durable cell anchorage through stable RGD immobilization, whereas controlled diffusion is more appropriate for antimicrobial peptides. For example, gelatin-chitosan antimicrobial peptide coatings can limit infection and support osteogenesis, with release tuned by lower MW and intermediate DD, which produce a less compact network (Savdenbekova et al., 2024; Ghimire et al., 2025; Elizalde-Cárdenas et al., 2024; Kiryk et al., 2025).

In bioinks for 3D bioprinting, chitosan can carry cells and biomolecules, while MW and DD govern rheology and network formation. Free peptides provide dynamic cues as the matrix remodels, whereas covalently tethered peptides preserve signal presentation during perfusion and extended culture. This stable presentation is often important for guiding lineage specification in pluripotent-cell bioinks (Hamdi et al., 2025; Kumar et al., 2025; Ding et al., 2025; Salamat et al., 2025; Elizalde-Cárdenas et al., 2024).

#### 4.2.4 Choosing the functionalization strategy

Free and bound peptides should be viewed as complementary rather than competing strategies. Hybrid biomaterials can immobilize part of the peptide at the surface to support immediate adhesion, while encapsulating the remaining fraction for longer-term signaling during degradation. This enables staged regulation of cellular responses, from early attachment to subsequent migration and differentiation. System design should therefore match chitosan MW and DD, material format, and peptide properties (Egorov et al., 2025; Elizalde-Cárdenas et al., 2024; Forero et al., 2023; Kiryk et al., 2025). These complementary roles are summarized in Figure 7.

**FIGURE 7:**
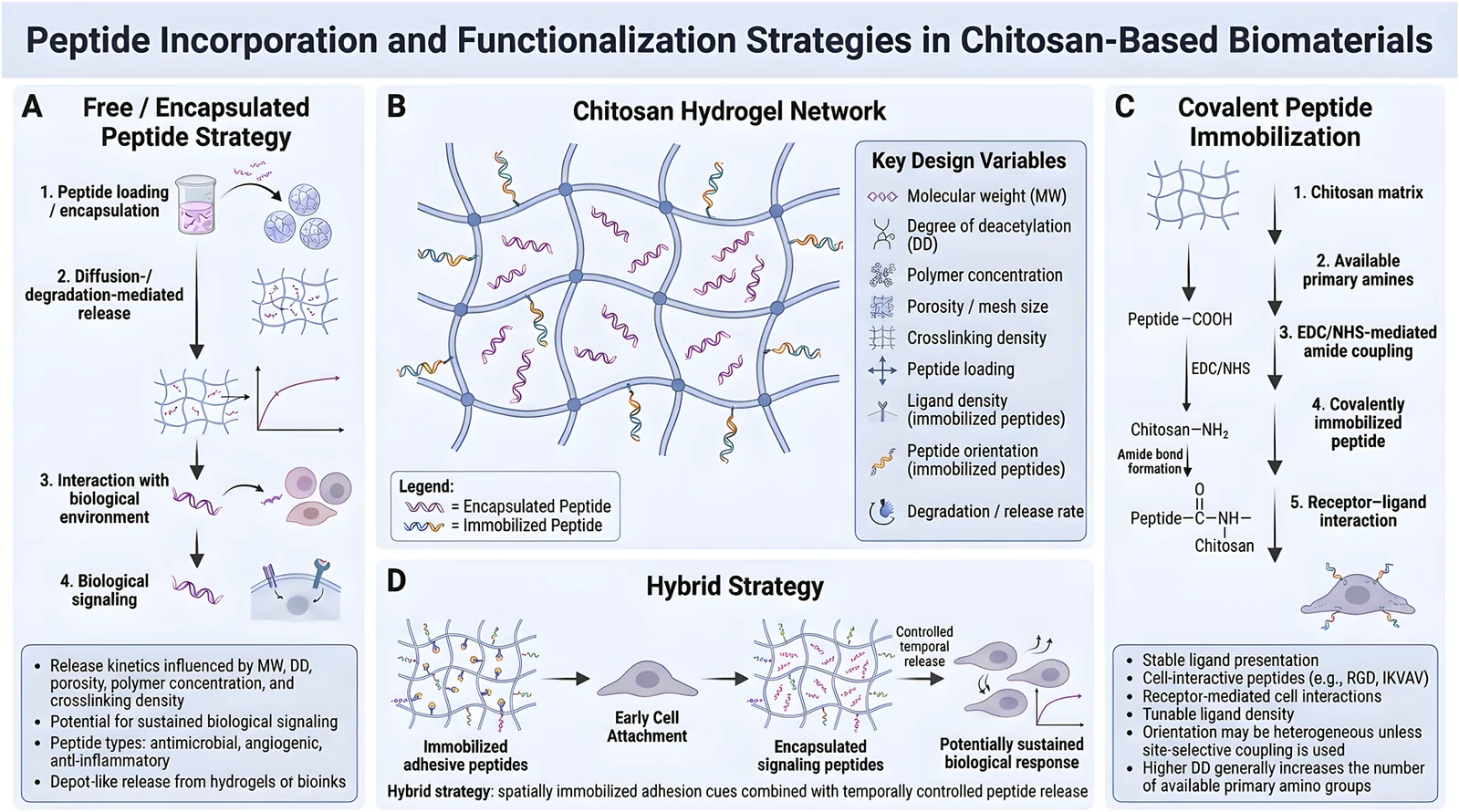
Peptide functionalization as a strategy to convert chitosan into a bio-instructive matrix. Because native chitosan lacks specific ECM-derived adhesion motifs, peptide incorporation can provide receptor-mediated cues for cell attachment, migration, angiogenesis, and tissue remodeling. Peptides may be physically entrapped for release-based activity or covalently immobilized to create more stable cell-instructive interfaces. This figure was prepared with the assistance of FigureLabs.ai.

### 4.3 Modification of chitosan by other polymers for tissue engineering applications

Chitosan properties are governed primarily by MW and DD. Higher DD increases the content of cationic amine groups, solubility in mild acids, and intermolecular interactions, whereas higher MW increases chain entanglement and viscosity. Together, these parameters shape complex formation, processing behavior, and biomolecule delivery (Egorov et al., 2025; Hamdi et al., 2025; Edo et al., 2024). Because neat chitosan can be brittle, mechanically limited, and slow or pH-dependent in gelation, it is commonly blended with natural or synthetic polymers to create composites with complementary or synergistic properties (Elizalde-Cárdenas et al., 2024). This section reviews such modifications across four tissue-engineering formats, namely, hydrogels, xerogels, implant coatings, and 3D-bioprinting bioinks, with emphasis on how co-polymer choice, DD, and MW govern processing, compatibility, and final material properties (Hamdi et al., 2025; Gholap et al., 2024).

#### 4.3.1 Chitosan-polymer hydrogels

Building on the DD- and MW-dependent gelation behavior established above, this section focuses specifically on how blending with a second polymer modifies chitosan hydrogel networks for soft-tissue engineering and bone filling (Salamat et al., 2025; Sauce-Guevara et al., 2025). Natural partners include gelatin, which forms polyelectrolyte complexes with protonated chitosan amines. When co-crosslinked, particularly in the presence of multivalent anions, gelatin and chitosan can form tougher hybrid networks while avoiding the dense precipitates often associated with chitosan-only crosslinking (Che et al., 2024; Guo et al., 2024; Salamat et al., 2025).

Anionic polysaccharides such as alginate and hyaluronic acid enable ionic crosslinking and add biofunctionality. Alginate, for example, crosslinks with Ca^2+^ and can stiffen chitosan-alginate complexes. Higher-DD chitosan strengthens these interactions through hydrogen bonding and ion pairing, whereas low-DD chitosan forms weaker networks unless additional crosslinking strategies are introduced (Hamdi et al., 2025; von Seggern et al., 2025; Sauce-Guevara et al., 2025).

Synthetic partners are used to tune mechanics and responsiveness. Chitosan/poly (vinyl alcohol) (PVA) double networks combine a rigid PVA crystalline phase that dissipates energy with a flexible chitosan network, resulting in highly tough hydrogels (Le Ba Thai et al., 2024; Bi et al., 2020). Polyethylene glycol (PEG) grafting increases chain mobility and can produce softer, injectable gels suitable for cartilage repair. Thermosensitive chitosan/poly (N-isopropylacrylamide) (PNIPAM) systems remain liquid at room temperature and gel at body temperature, enabling *in situ* delivery. In these systems, MW and DD influence pre-gel viscosity, gelation temperature, and mesh size (Egorov et al., 2025; Salamat et al., 2025).

In general, natural additives such as gelatin and collagen contribute cell-adhesive sites and softer matrices, whereas synthetic partners such as PVA, PEG, and PNIPAM provide mechanical reinforcement or stimulus responsiveness. Moderate MW, approximately 50–200 kDa, often provides a useful balance between processability and mechanical stability (Hamdi et al., 2025; Salamat et al., 2025; Sauce-Guevara et al., 2025).

#### 4.3.2 Chitosan-polymer xerogels

Xerogels are dried polymer networks, typically porous freeze-dried sponges, used as scaffolds for skin, bone, nerve, and cartilage applications (Tupe et al., 2024). Pure freeze-dried chitosan sponges are biocompatible and porous, but they are usually mechanically weak and can degrade rapidly. For this reason, chitosan is commonly blended with structural polymers before drying (Elizalde-Cárdenas et al., 2024; Tupe et al., 2024).

Collagen and ECM-derived glycoproteins improve tissue mimicry. Lyophilized collagen-chitosan sponges show increased resistance to enzymatic degradation after mild crosslinking while retaining cytocompatibility. In such systems, chitosan can act as a physical or chemical crosslinking component that extends *in vivo* residence time, whereas collagen or gelatin supports cell adhesion and infiltration. Composites incorporating fibrinogen-binding domains have also been reported to accelerate wound closure and vascularization in full-thickness defects through combined hemostatic and antimicrobial effects (Mawazi et al., 2024; Ding et al., 2025; Elizalde-Cárdenas et al., 2024; Tupe et al., 2024).

Other natural partners, including silk fibroin, elastin, alginate, and glycosaminoglycans, modulate scaffold mechanics and pore architecture. Silk fibroin and elastin are particularly useful for increasing tensile strength and elasticity (Tupe et al., 2024). DD strongly influences microstructure. High-DD chitosan forms stronger interpolymer and hydrogen bonds, leading to smaller pores, stiffer matrices, higher crystallinity, lower swelling, and greater protein adsorption. These features may be useful for nerve conduits and load-bearing implants. Lower-DD chitosan is more hydrophobic and can produce larger, more open pores, which may be advantageous for soft tissues requiring rapid cell infiltration (Egorov et al., 2025; Hamdi et al., 2025; von Seggern et al., 2025).

Synthetic partners add durability and processing flexibility. Polyesters such as PLGA and polycaprolactone (PCL) provide slower degradation and improved mechanical strength, while chitosan contributes hydrophilicity and biofunctionality. Because pure chitosan is difficult to electrospin, co-spinning with poly (ethylene oxide) or PCL is often used to obtain nanofiber mats. In electrospun chitosan/poly (ethylene oxide) scaffolds, approximately 85% DD balanced flexibility and osteoblast-like cell support, whereas very high DD increased brittleness and very low DD reduced cell attachment (Hamdi et al., 2025; Elizalde-Cárdenas et al., 2024; Tupe et al., 2024).

#### 4.3.3 Chitosan-based implant coatings

Composite coatings combine the bioactivity and antibacterial potential of chitosan with the mechanical or chemical stability of a partner polymer, addressing the limited adhesion and softness of pure chitosan films (Teixeira-Santos et al., 2021; Ghimire et al., 2025). In polyelectrolyte multilayers, layer-by-layer assembly alternates cationic chitosan with an anionic polymer such as PAA or alginate. Chitosan/PAA multilayers incorporating chlorhexidine on silicon and titanium achieved durable and sustained antibacterial activity. In these systems, electrostatic bonding is tuned by pH and DD. Higher DD generally produces thicker, more crosslinked films with higher drug-binding capacity, whereas lower DD forms looser layers with faster release (Egorov et al., 2025; von Seggern et al., 2025; Savdenbekova et al., 2024).

In blended single-layer coatings, higher DD strengthens binding to anionic partners and stiffens the film. However, polymer ratios must be optimized to obtain uniform layers. PEG grafting can introduce antifouling behavior, but excessive PEG content may weaken cell-adhesive and antibacterial effects. For bone-contacting coatings, nano-hydroxyapatite embedded in chitosan/polymer matrices can enhance osteoblast differentiation by combining organic and inorganic cues. Higher-DD chitosan may further support this function by chelating Ca^2+^ and binding hydroxyapatite more strongly (Egorov et al., 2025; Ghimire et al., 2025; Piszko et al., 2024).

Coatings should be thin but durable. Medium-MW chitosan, approximately 50–300 kDa, can form coherent and crack-free films, whereas very high MW may hinder uniform application. Near-complete DD can also reduce solubility and complicate coating preparation (Egorov et al., 2025; Ghimire et al., 2025). Properly designed multilayer or blended coatings can protect implants, deliver therapeutics, and promote tissue integration, including increased osteoblast attachment and new bone formation on chitosan-coated titanium (Savdenbekova et al., 2024; Ghimire et al., 2025; Villegas et al., 2022).

#### 4.3.4 Chitosan-polymer bioinks for 3D bioprinting

Bioinks are biomaterial formulations, often hydrogel precursors, used in 3D bioprinting. A successful bioink must balance printability, shear-thinning rheology, post-printing fidelity, cytocompatibility, and gelation kinetics (Mathur et al., 2025). Chitosan is attractive because of its biocompatibility and antibacterial potential, but it is difficult to print alone because it is soluble mainly under acidic conditions and can gel or precipitate during neutralization. Therefore, it is usually blended with polymers that stabilize printing and provide complementary crosslinking mechanisms (Salamat et al., 2025).

A common strategy is to pair chitosan with thermoresponsive or ionically crosslinkable polymers that enable rapid post-extrusion stabilization. Chitosan-gelatin-alginate inks use gelatin to support thermoreversible filament formation, chitosan to provide biofunctionality, and alginate to enable rapid Ca^2+^-mediated ionic crosslinking. Methacrylated gelatin can additionally provide photocrosslinking (Sauce-Guevara et al., 2025; Mathur et al., 2025; Reberte et al., 2025).

Chitosan is also combined with synthetic polymers such as Pluronic F127 or poly (ethylene glycol) diacrylate (PEGDA). Methacrylated chitosan co-printed with PEGDA forms photocrosslinked interpenetrating networks that combine chitosan-derived adhesion and biodegradability with rapid PEGDA curing. Cellulose nanofibers can also be incorporated to provide shear-thinning behavior followed by ionic crosslinking (Ding et al., 2025; Mathur et al., 2025).

Performance depends strongly on MW and DD. Higher MW increases viscosity, which helps prevent cell settling and maintain filament shape. However, excessive MW, particularly above approximately 500 kDa, can impair extrusion and increase shear stress on cells. For this reason, many bioinks use chitosan in the approximate range of 100–300 kDa. High DD strengthens interactions with polyanions, supporting faster self-setting and better shape fidelity, but it can also cause premature gelation in the nozzle. Lower DD delays gelation and provides more working time, although often at the cost of reduced network strength (Hamdi et al., 2025; Salamat et al., 2025; Mathur et al., 2025; Reberte et al., 2025).

Natural partners such as gelatin and alginate provide biocompatibility and mild crosslinking triggers. Synthetic components such as Pluronic, PEGDA, and modified chitosan provide more precise control over gelation and mechanics. Chitosan may also contribute antimicrobial support, but this function should be validated in the final printed matrix rather than inferred from the presence of chitosan alone (Ding et al., 2025; Salamat et al., 2025; Elizalde-Cárdenas et al., 2024; Reberte et al., 2025).

#### 4.3.5 Synthesis of composite design principles

Across hydrogels, xerogels, coatings, and bioinks, complementary polymers help offset the main limitations of chitosan, including weak mechanics and processing challenges. Natural polymers such as collagen, gelatin, and alginate improve cell interactions and bioactivity, whereas synthetic polymers such as PVA, PEG, PNIPAM, PLA, and PCL provide strength, stability, and more controlled gelation (Salamat et al., 2025; Elizalde-Cárdenas et al., 2024; Tupe et al., 2024; Mathur et al., 2025).

DD and MW remain the two central design variables. Higher DD increases reactivity and network strength, but excessive DD can accelerate gelation, increase brittleness, or reduce solubility. Higher MW reinforces networks through chain entanglement, but overly high MW can hinder mixing, film formation, or extrusion. These parameters should therefore be selected according to the intended application. For example, approximately 80%–90% DD combined with medium MW may be suitable for tough and adhesive bone-graft hydrogels, whereas slightly lower DD or MW may be preferable for printable bioinks (Egorov et al., 2025; Hamdi et al., 2025; von Seggern et al., 2025; Salamat et al., 2025; Reberte et al., 2025).

Mastering the interplay between DD, MW, and the selected polymer partner enables the design of finely tuned biomaterials for regenerative medicine. Composite strategies can produce robust double-network hydrogels, cell-friendly porous sponges, infection-resistant coatings, and printable tissue-mimicking bioinks (Egorov et al., 2025; Koirala et al., 2025; Mawazi et al., 2024; Elizalde-Cárdenas et al., 2024).

## 5 Ethical, regulatory, and translational considerations

Ethical and translational analysis of chitosan-based regenerative platforms should focus on preventable scientific harms rather than on generic statements about animal origin or natural materials. As discussed in the sections on raw-material purity/endotoxin control and on growth-factor, drug, and extracellular-vesicle functionalization above, the recurring risks are concrete and include under-characterized animal-derived inputs, residual proteins and allergens, endotoxin and β-glucan contamination, premature claims of biocompatibility or regeneration, and unvalidated extracellular-vesicle (EV) or cell potency. These concerns are not abstract, because they directly affect patient safety, reproducibility, animal welfare, and public trust (Reay et al., 2023; International Society for Stem Cell Research, 2021; Food and Drug Administration, 2023).

A central point is that a biomaterial-only chitosan dressing, an antibiotic-loaded scaffold, a BMP-2-loaded hydrogel, an MSC-seeded construct, and an EV-loaded matrix are ethically and regulatorily distinct products. The ethical and evidentiary burden increases as the intended mode of action shifts from material-based function toward pharmacological, cellular, or vesicle-mediated mechanisms. Such platforms should therefore not be described simply as chitosan biomaterials (Food and Drug Administration, 2022; European Medicines Agency, 2020).

### 5.1 From biomaterial to combination product: Why classification matters

Regulatory classification depends on the components, intended use, and primary mode of action. It should be addressed early, because misclassification can lead to inadequate safety testing, unrealistic development timelines, and premature clinical claims. In the United States, combination products comprise two or more regulated components, including drug-device, biologic-device, or drug-device-biologic combinations, as well as cell-seeded or biologic-coated devices. Accordingly, a chitosan scaffold combined with BMP-2, an antibiotic, viable cells, or EVs requires combination-product analysis rather than device-only logic, with the primary mode of action guiding the regulatory pathway (Food and Drug Administration, 2022; ECFR, 2026).

In the European Union, advanced therapy medicinal product frameworks cover gene therapy, somatic-cell therapy, tissue-engineered products, and combined ATMPs. A chitosan scaffold carrying viable cells may therefore fall under tissue-engineered product or combined-ATMP rules. EV products raise analogous concerns related to source, manufacturing, characterization, potency, and safety, even where they do not map cleanly onto existing categories (International Society for Stem Cell Research, 2021; Lener et al., 2015; European Medicines Agency, 2020; European Medicines Agency, 2026). Authors should distinguish scientific category from legal category, avoid jurisdiction-independent regulatory claims, and not imply that the food-use history of chitosan reduces the evidentiary burden for a combination product.

### 5.2 Risk-based biocompatibility and product-specific evaluation

Biocompatibility is a property of a specific final product, not of the base polymer. FDA guidance on ISO 10993-1 frames biological evaluation within a risk-management process that considers the nature and duration of body contact, materials, manufacturing, and sterilization (Food and Drug Administration, 2023). The same chitosan may therefore require very different testing packages depending on whether it is used as a topical dressing, absorbable implant, injectable hydrogel, blood-contacting particle, drug depot, or cell- or EV-loaded scaffold. Relevant endpoints may include cytotoxicity, sensitization, irritation, systemic toxicity, implantation, hemocompatibility, genotoxicity, and degradation products, depending on the intended use.

For animal-derived chitosan, ISO 22442-style provenance and sourcing logic should inform risk management (Food and Drug Administration, 2019). For cell- or EV-containing products, scaffold biocompatibility is necessary but not sufficient. The biological component requires its own framework for identity, purity, potency, and safety (International Society for Stem Cell Rese arch, 2021; Welsh et al., 2024).

### 5.3 Reproducibility and research integrity

Reproducibility failures are themselves an ethical problem because they waste animals, misdirect funding, and risk translating artifacts of contamination, formulation, or assay design into clinical narratives. The recurrent weaknesses discussed throughout the raw-material and functionalization sections above, including missing MW and DD data, unreported acetylation pattern, unknown endotoxin or β-glucan status, undocumented sterilization, weak comparators, and EV misclassification, should be treated as research-integrity issues rather than minor reporting gaps (Patrulea et al., 2015; Reay et al., 2023; Welsh et al., 2024). These risk categories, their translational significance for chitosan specifically, and the corresponding minimum expectations for responsible reporting are summarized in Table 6.

**TABLE 6:** Ethical and translational risk-control matrix for chitosan-based regenerative platforms.

Translation issue	Why it matters for chitosan	Minimum expectation	Key sources
Animal-derived provenance	Crustacean sources may vary in species, mineral burden, residual proteins, and collection/processing controls	Traceable source, validated deproteinization/demineralization, residual-protein and ash/metals specifications, change-control plan	(Food and Drug Administration, 2019; Patrulea et al., 2015; Waibel et al., 2011)
Endotoxin and β-glucan	Endotoxin can dominate macrophage/PBMC cytokine readouts; fungal or plant-derived inputs can cause β-glucan-related assay interference	Validated extraction, spike recovery, assay interference testing, rFC or recombinant reagent strategy when β-glucan interference is plausible	(Reay et al., 2023; Banach-Kopeć et al., 2022; European Directorate for the Quality of Medicines and HealthCare, 2023; United States Pharmacopeia, 2024)
Cell-loaded constructs	Adding viable cells changes the product from a biomaterial to a cell/scaffold product with donor, potency, tumorigenicity, and long-term safety issues	Donor consent and screening, traceability, release criteria, genetic stability, potency in the final scaffold-cell configuration	(International Society for Stem Cell Research, 2021; Food and Drug Administration, 2023)
EV-loaded constructs	EV preparations are heterogeneous and may contain non-vesicular proteins, lipoproteins, serum contaminants, or FBS-derived EVs	Source, isolation, characterization, purity, potency, released-fraction testing, and culture-medium controls consistent with current extracellular vesicle guidelines.	(Théry et al., 2018; Welsh et al., 2024; Urzì et al., 2022; Mannerström et al., 2019)
Regulatory classification	Chitosan plus drug, growth factor, EV, or cells may be regulated as a combination product or advanced-therapy product rather than as a simple device	Early primary-mode-of-action analysis, jurisdiction-specific regulatory strategy, final-product biological evaluation, GMP-compatible manufacturing	(Food and Drug Administration, 2022; ECFR, 2026; European Medicines Agency, 2020; European Medicines Agency, 2026)

A defensible endotoxin strategy should specify the assay type. Recombinant factor C may be considered as an animal-free alternative, particularly when β-glucan interference is plausible (European Directorate for the Quality of Medicines and HealthCare, 2023; United States Pharmacopeia, 2024; Gorman, 2020). The corresponding discipline is linguistic as much as experimental. Authors should avoid describing chitosan as biocompatible, pro-regenerative, or complement-activating as a universal property. Instead, they should state that a defined material, with defined characterization, produced a defined effect under defined conditions, with relevant confounders made explicit.

## 6 Conclusion

Chitosan has earned sustained attention in regenerative medicine because it can be processed into hydrogels, porous scaffolds, films, coatings, nanoparticles, bioinks, and composite matrices while offering chemically accessible amine groups, pH-dependent charge, enzymatic degradability, wet-tissue interactions, and cargo-binding capacity. The evidence reviewed here supports chitosan as a useful biomaterial platform, but only when it is described as a defined material in a defined formulation. General statements that chitosan is biocompatible, antimicrobial, hemostatic, immunomodulatory, or regenerative are scientifically weak unless they are bounded by DD, MW, pattern of acetylation, purity, endotoxin and β-glucan status, formulation format, dose, exposure route, and biological model.

The most important translational lesson is that chitosan performance is interfacial and product-specific. Cells rarely encounter pristine polymer; they encounter a dynamic biointerface shaped by protein adsorption, mucus or blood components, salts, pH, degradation products, residual reagents, sterilization effects, and device architecture. This is particularly important for immune and blood-contacting endpoints, where endotoxin, β-glucan, complement binding, coagulation, platelet activation, and protein corona formation can substantially alter the interpretation of mechanistic studies. Similarly, antimicrobial activity demonstrated for soluble protonated chitosan should not be assumed for crosslinked hydrogels, xerogels, coatings, composites, or bioinks unless the final formulation is tested against relevant organisms under relevant pH, ionic strength, serum, and biofilm conditions.

For advanced platforms, the evidentiary threshold increases. Growth-factor-loaded chitosan systems should demonstrate loading, release in relevant media, post-release bioactivity, dose realism, and sterilization stability. Drug-loaded systems should integrate antimicrobial or anti-inflammatory efficacy with host-cell compatibility and tissue-repair outcomes. EV-loaded chitosan matrices should use terminology consistent with current extracellular vesicle guidelines, characterize both input and released EV fractions, control for non-vesicular contaminants and FBS-derived material, and establish release-normalized potency. Cell-loaded constructs require donor traceability, cell-product characterization, potency in the final scaffold-cell configuration, and long-term safety considerations.

Future progress will depend less on adding more chitosan formulations and more on improving comparability, quality control, and regulatory-aware design. Minimum reporting should include source, lot, DD, MW distribution, polydispersity, pattern of acetylation where feasible, residual protein, ash/metals, endotoxin testing with interference controls, β-glucan strategy when relevant, solvent and pH history, crosslinking chemistry, residual reagents, sterilization method, storage conditions, degradation testing, and final-product biological evaluation. Clinical translation will require clinically meaningful comparators, mechanism-linked potency assays, scalable manufacturing, GMP-compatible inputs, and early classification of device, combination-product, ATMP, or biologic-like regulatory pathways.

Chitosan should therefore be positioned not as a universally regenerative natural polymer, but as a tunable aminopolysaccharide platform whose value lies in disciplined material definition, formulation-specific biological testing, and integration with realistic translational constraints. With standardized characterization, evidence-based claim language, and product-specific regulatory strategy, chitosan-based biomaterials can continue to contribute to regenerative medicine while avoiding the reproducibility and overstatement problems that have limited many promising preclinical technologies.

## Glossary

DD: degree of deacetylation
MW: molecular weight
ECM: extracellular matrix
EVs: extracellular vesicle
BMP: bone morphogenetic protein
VEGF: vascular endothelial growth factor
PDGF: platelet-derived growth factor
FGF: fibroblast growth factor
NGF: nerve growth factor
TNF: tumor necrosis factor
RGD: arginine-glycine-aspartate
SIKVAV: serine-isoleucine-lysine-valine-alanine-valine
MSC: mesenchymal stromal/stem cell
PBMC: peripheral blood mononuclear cell
LPS: lipopolysaccharide
LAL: Limulus amebocyte lysate
rFC: recombinant factor C
ISO: International Organization for Standardization
GMP: Good Manufacturing Practice
FDA: U.S. Food and Drug Administration
ATMP: advanced therapy medicinal product
PBS: phosphate-buffered saline
FBS: fetal bovine serum
PEG: polyethylene glycol
PEGDA: poly(ethylene glycol) diacrylate
PVA: poly(vinyl alcohol)
PNIPAM: poly(N-isopropylacrylamide)
PCL: polycaprolactone
PLA: polylactic acid
PLGA: poly(lactic-co-glycolic acid)
TPP: tripolyphosphate
EDC: 1-ethyl-3-(3-dimethylaminopropyl)carbodiimide
NHS: N-hydroxysuccinimide
EO: ethylene oxide
